# Combining Quantitative Genetic Footprinting and Trait Enrichment Analysis to Identify Fitness Determinants of a Bacterial Pathogen

**DOI:** 10.1371/journal.pgen.1003716

**Published:** 2013-08-22

**Authors:** Travis J. Wiles, J. Paul Norton, Colin W. Russell, Brian K. Dalley, Kael F. Fischer, Matthew A. Mulvey

**Affiliations:** 1 Division of Microbiology and Immunology, Pathology Department, University of Utah School of Medicine, Salt Lake City, Utah, United States of America; 2 Huntsman Cancer Institute, University of Utah School of Medicine, Salt Lake City, Utah, United States of America; 3 ARUP Laboratories, Salt Lake City, Utah, United States of America; The University of Texas Health Science Center at Houston, United States of America

## Abstract

Strains of **Ex**traintestinal **P**athogenic *Escherichia* ***c*** *oli* (ExPEC) exhibit an array of virulence strategies and are a major cause of urinary tract infections, sepsis and meningitis. Efforts to understand ExPEC pathogenesis are challenged by the high degree of genetic and phenotypic variation that exists among isolates. Determining which virulence traits are widespread and which are strain-specific will greatly benefit the design of more effective therapies. Towards this goal, we utilized a quantitative genetic footprinting technique known as transposon insertion sequencing (Tn-seq) in conjunction with comparative pathogenomics to functionally dissect the genetic repertoire of a reference ExPEC isolate. Using Tn-seq and high-throughput zebrafish infection models, we tracked changes in the abundance of ExPEC variants within saturated transposon mutant libraries following selection within distinct host niches. Nine hundred and seventy bacterial genes (18% of the genome) were found to promote pathogen fitness in either a niche-dependent or independent manner. To identify genes with the highest therapeutic and diagnostic potential, a novel **T**rait **E**nrichment **A**nalysis (TEA) algorithm was developed to ascertain the phylogenetic distribution of candidate genes. TEA revealed that a significant portion of the 970 genes identified by Tn-seq have homologues more often contained within the genomes of ExPEC and other known pathogens, which, as suggested by the first axiom of molecular Koch's postulates, is considered to be a key feature of true virulence determinants. Three of these Tn-seq-derived pathogen-associated genes—a transcriptional repressor, a putative metalloendopeptidase toxin and a hypothetical DNA binding protein—were deleted and shown to independently affect ExPEC fitness in zebrafish and mouse models of infection. Together, the approaches and observations reported herein provide a resource for future pathogenomics-based research and highlight the diversity of factors required by a single ExPEC isolate to survive within varying host environments.

## Author Summary

Antibiotic resistance is an increasingly serious problem, especially among pathogenic strains of *Escherichia coli* that cause urinary tract infections, sepsis and meningitis. It is important to obtain a more comprehensive genome-wide understanding of bacterial virulence because it has the potential to uncover novel and alternative therapeutic targets. Therefore, we probed the genome of a pathogenic *E. coli* isolate using transposon mutagenesis, deep sequencing and comparative pathogenomics in an effort to define its virulence gene repertoire. Using this multilayered approach in combination with high-throughput zebrafish infection models, we identified hundreds of genes that affect pathogen fitness during localized and/or blood-borne infections. We also developed a bioinformatics-based method to systematically sift through our datasets for genes that are broadly conserved among an assortment of pathogenic species. Follow-up analysis of several pathogen-associated candidate genes using zebrafish and mouse infection models highlighted the capacity of our approach to identify novel fitness determinants. The results from this study are available via an interactive online data viewer (http://pathogenomics.path.utah.edu/F11_TnSeq/) so that investigators can more effectively search and utilize these findings.

## Introduction

Within the *Escherichia coli* lineage there are several distinct virulent subgroups that are principally classified by an ability to cause a common set of diseases. One specific subgroup, **Ex**traintestinal **P**athogenic ***E. c*** *oli* (ExPEC), is typically thought to be a benign inhabitant of the lower intestinal tract of warm-blooded vertebrates. However, outside this niche, ExPEC strains have the ability to persist in an array of secondary host-associated habitats where they can cause urinary tract infections (UTIs), meningitis, bacteremia and sepsis in both humans and domesticated animals [1], [2], [3], [4]. The combined medical, agricultural and economic burden of ExPEC-related diseases is likely to increase as antibiotic resistance spreads [1], [5], [6]. Delineation of the genetic elements utilized by ExPEC to infect such a diverse spectrum of host niches and cause disease promises to advance our understanding of pathogen evolution and behavior while also highlighting more effective strategies to combat these pervasive opportunistic pathogens.

Despite colonizing similar ecological niches, ExPEC isolates can differ by 20–30% of their respective gene inventories and to date, a specific and ubiquitous molecular trait or function exclusive to this cohort has not been characterized [7], [8]. Without knowledge of a unifying ExPEC-associated feature, the development of broad-spectrum therapeutic strategies is made exceptionally difficult [9]. Previous work indicates that individual ExPEC strains appear to have evolved distinct genetic repertoires that promote unique and at times subtle fitness advantages during colonization of specific host environments [10], [11]. It is becoming clear that the working definition of ExPEC is more multigenic in nature. Further complicating the search for ExPEC-associated traits is the 30–40% of genes within their genomes that still require functional annotation [7]. This problem will likely persist as genome sequencing continues to outstrip rates of experimental characterization. Consequently, there is a pressing need to develop methods that mesh high-throughput genomics with context-based functional observations—a notion that is becoming increasingly appreciated [12], [13]. To this end, we adapted a previously described transposon mutagenesis technique known as ‘Tn-seq’ to identify genes that promote pathogen fitness during colonization of a vertebrate host [14], [15].

The use of transposons to conduct unbiased forward genetic screens in bacteria has provided many avenues for investigation over the last two decades [16], [17], [18]. However, these approaches are, for the most part, time and labor intensive and often lack reliable quantitative metrics and sampling depth to identify genes of interest for retrospective follow-up. On the other hand, transposon mutagenesis as a tool is quite amenable to innovation [19], [20], [21]. Tn-seq, also known as ‘INSeq’, utilizes deep sequencing to monitor the composition of insertion variants within bulk mutant pools [14], [15]. This is accomplished through use of a modified *mariner* transposon that contains recognition sites within the two distal inverted repeats that are specific for the restriction enzyme MmeI. Cleavage of DNA by this enzyme occurs 18–20 base pairs from its recognition site, such that excision of the transposon by MmeI captures flanking genomic sequences. These sequences serve as tags that can be used to track the relative abundance of distinct insertion variants within heterogenetic mutant libraries. This technique ultimately allows the fitness of thousands of mutant variants to be quantified simultaneously following exposure to specific selective pressures. By comparing the composition and abundance of mutants before and after passage through selective conditions, such as within a host organism, a genome-wide map of loci that are important for bacterial fitness can be assembled.

The screening approach reported here involved injection of 48 h post-fertilization (hpf) zebrafish embryos with transposon-mutagenized pools of ExPEC derived from a single parent isolate. Embryonic zebrafish are particularly useful for this type of selection screen because their vertebrate physiology closely matches that of humans, and they have proven useful for the identification and characterization of virulence determinants that enable ExPEC to survive within host niches [10], [11], [22], [23]. Of particular importance, zebrafish embryos possess many of the same innate defenses that mammalian hosts rely upon to resist ExPEC (e.g. phagocytes, complement and antimicrobial peptides) [10], [24], [25], [26]. This attribute underscores the ability of the zebrafish host to mirror a set of overlapping selective pressures that ExPEC naturally encounter. Zebrafish embryos can be used to model both localized and systemic infections and the complete recovery and enumeration of bacteria from within the entire host is relatively straightforward [10], [11]. Through three independent biological experiments employing Tn-seq we identified 970 genes (∼18% of gene content) that promote the competitive fitness of ExPEC during either localized or systemic infections. A large number of these genes have unknown functions, while many others have reported roles in iron transport, bacterial secretion, two-component signaling and metabolism. To focus on genes that are likely important to ExPEC pathogenesis specifically, we devised a gene ontology-like method called ‘TEA’ (**T**rait **E**nrichment **A**nalysis), that enabled us to organize candidate genes according to their association with certain bacterial lineages or groups of bacteria that are phenotypically similar. The data presented in this report indicate that TEA, in combination with Tn-seq, provides an effective, streamlined approach for identifying biologically relevant fitness and virulence determinants that are employed by ExPEC within various host environments. Datasets generated in this study have been made freely available through a curated and searchable web-based data viewer (http://pathogenomics.path.utah.edu/F11_TnSeq/).

## Results

### Mutant library construction and screen design

**Figure 1:**
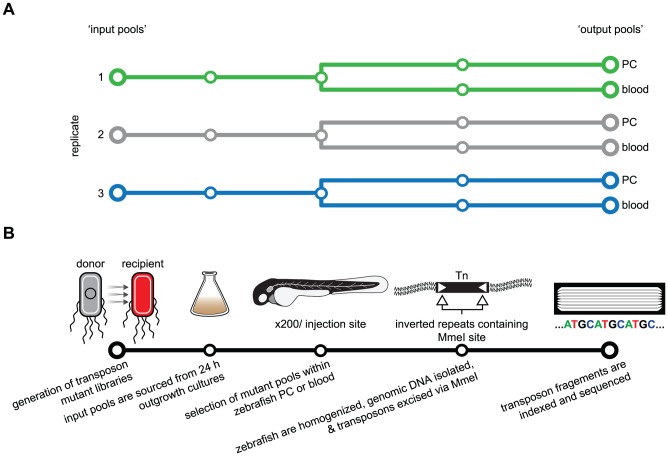
Schematic of the Tn-seq selection screen used to identify genes necessary for fitness in a zebrafish infection model. (A) Three selection screens were performed (green, grey and blue tracks) using three ‘input’ pools to generate a total of six ‘output’ pools. (B) For each replicate screen, an independent transposon mutant library was created. Expansion of mutant libraries from frozen stocks was done at 37°C for 24 h to produce input pools and starting inoculum. Approximately 200 zebrafish embryos were injected in the pericardial cavity (PC) or circulation valley (CV, blood) with approximately 3,000 mutant bacteria. After a selection period of 20 h, zebrafish were homogenized to facilitate recovery and extraction of total DNA. The restriction enzyme MmeI was used to excise transposons and flanking F11 genomic DNA for analysis by deep sequencing.

We previously described the use of the embryonic zebrafish as a surrogate host to study genotype-phenotype relationships of a variety of human and non-human ExPEC isolates [10], [11]. During this initial work we observed that the human cystitis (bladder infection) isolate F11 is particularly adept at growing within and eliciting death of zebrafish during either localized or systemic infections. Elucidation of the virulence gene repertoire (i.e. genetic determinants that promote pathogenic behaviors such as colonization or destruction of host tissues) of this ExPEC strain using Tn-seq provides a starting point for future comparative pathogenomics studies. Figure 1 presents an outline detailing the negative selection screen carried out in this study.

Transposon mutagenesis was accomplished by using the previously described pSAM vector and conjugation [15]. This plasmid, which contains a *mariner* transposon flanked by MmeI modified inverted repeats and the *himar*1C9 transposase, was retrofitted with an *E. coli* compatible antibiotic resistance cassette and promoter elements to generate pSAM-Ec (Figure S1). Multiple, independent mating events were performed to assemble three transposon mutant libraries to be used in three separate replicate screens (Figure 1A). It was determined that each pool contained a minimum of 50,000 distinct insertion variants by enumerating selectable, transposon positive colonies immediately following conjugation when mutant siblings are minimal. Successful transposition of single inserts into individual bacterial chromosomes was verified by detection of the transposon by Southern blot analysis (Figure S2). Because the cost of a pre-screen sequence analysis was prohibitive, we utilized a more economical means to confirm the diversity and saturation of mutant pools. The frequency of variants defective for lactose utilization, curli production and glycogen storage was determined using standard colorimetric plating assays (Figure S2), which demonstrated that our transposon mutant libraries were sufficiently saturated and ready for *in vivo* screening. Post-hoc sequencing later confirmed that our libraries contained 60,000 to 80,000 mutant variants each, corresponding to an insertion event approximately every 75 bp within the F11 chromosome (Table S1).

For each independent biological experiment, a single mutant library was cultivated from a frozen stock in M9 minimal media overnight at 37°C (Figure 1B). During this initial outgrowth period, variants with insertions that disabled genes critical for normal replication within broth culture were likely reduced in abundance or completely eliminated. Prior to injection of 48 hpf zebrafish embryos, a 1 ml aliquot of each overnight culture was pelleted, washed and suspended in phosphate buffered saline (PBS) at the desired density. A second aliquot was pelleted and stored as a reference ‘input’ population for later sequencing and comparative analyses with ‘output’ populations. A dose of approximately 3,000 colony forming units (CFU) from each mutant library was injected into either the pericardial cavity (PC) or into the blood via the circulation valley (CV) of zebrafish embryos (200 embryos per niche per experiment were used to ensure adequate sampling of mutant variants) (Figure S3). Injection of the PC provides a model of localized tissue infection, whereas injection into the CV results in rapid dissemination of ExPEC via the bloodstream, modeling bacteremia or sepsis-like infections [10], [11]. We previously determined that a variety of ExPEC isolates have the capacity to grow unchecked within the PC, while only a subset are able to persist and multiply within the bloodstream. Presumably, the microenvironments encountered by ExPEC following injection into the zebrafish circulatory system are more challenging than the PC with respect to nutrient availability, host defenses or other as-yet-undefined factors [10], [11]. F11 is able to survive and replicate within both the PC and blood. Once injected, selection proceeded for 18 to 20 h—a timeframe in which the mutant F11 population grows to between 10^4^ and 10^6^ CFU/embryo and elicits death in ∼40–60% of the animals (Figure S4). Total genomic DNA of the surviving F11 variants was recovered from batch-homogenized fish (alive and dead) to produce output pools. In parallel, equal amounts of genomic material from input and corresponding output pools were digested using the MmeI restriction enzyme followed by enrichment of transposon-containing fragments with flanking genomic sequences via gel electrophoresis. The fragments were then excised and prepared for indexed sequencing on a single lane of an Illumina HiSeq 2000. General sequence-based features of each mutant pool, including number of detected mutant variants, sequencing depth, saturation and insertional bias, are summarized in Table S1.

### Genetic footprinting by Tn-seq reveals distinguishable gene sets that have distinct functional compositions within the ExPEC genome

#### Gene insertion tolerance after passage in broth culture

**Figure 2:**
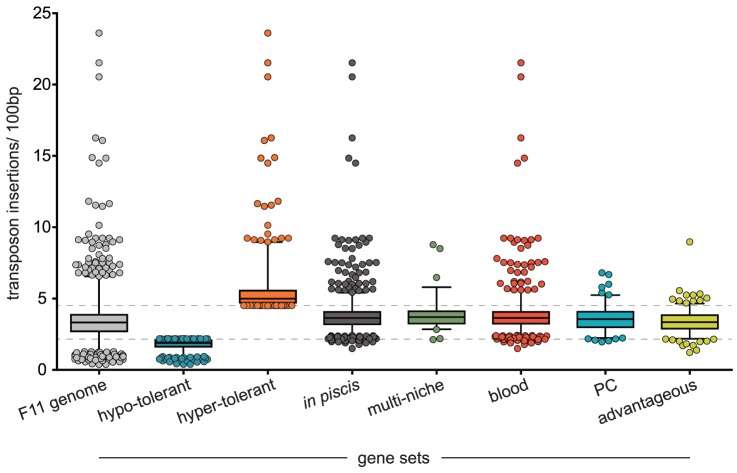
Transposon insertion rates for loci within different gene sets. From each input pool (1, 2 and 3) an average number of insertions detected per 100 bp was calculated for each annotated region within the F11 genome. Box plots indicate median and interquartile ranges with whiskers extending to the 1^st^ and 99^th^ percentiles. Dashed lines mark the upper and lower boundaries for hypo and hyper-tolerant gene sets, respectively. Gene set sizes: F11 genome - 5,312; hypo-tolerant - 609; hyper-tolerant - 431; *in piscis* - 970; multi-niche - 76; blood - 772; PC - 122; advantageous - 227. Note, the insertion rates reported here are expected to be overestimated—please refer to Methods section ‘*Candidate Identification*’ for a complete description of how the number of transposon insertion events was determined.

**Figure 3:**
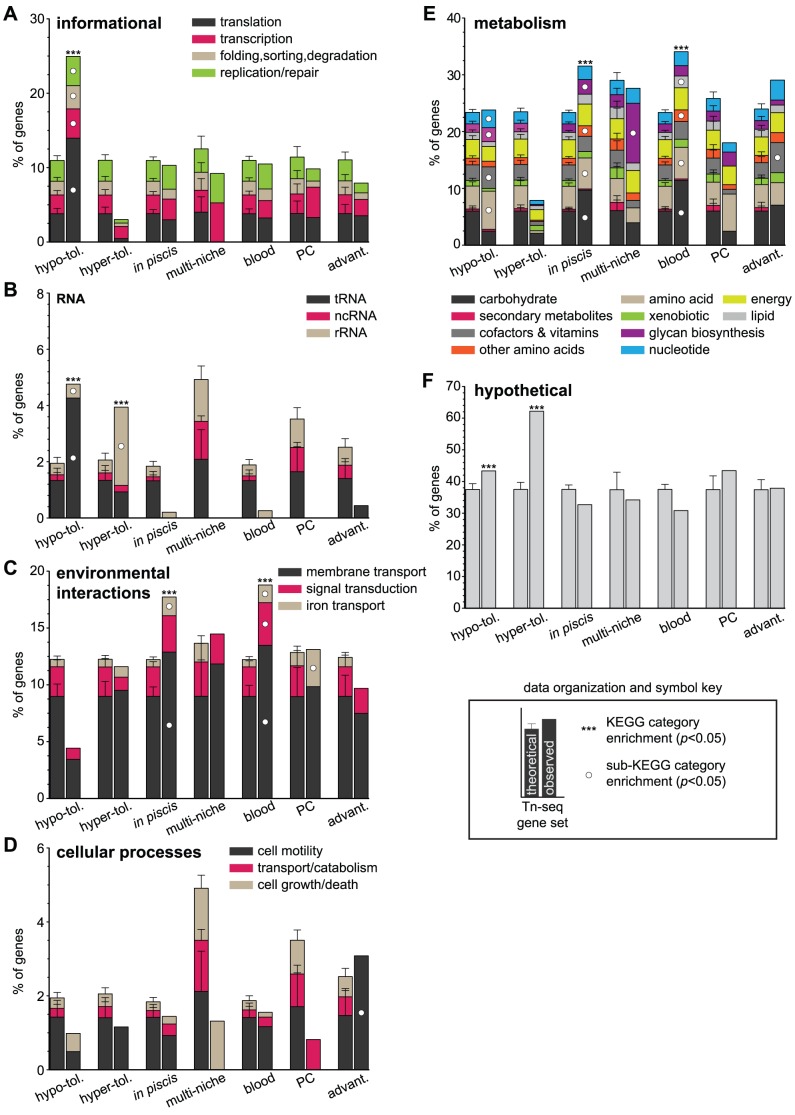
Functional KEGG categories enriched within gene sets. To orient the reader to graph organization and symbols, a key is provided in the lower right corner. The cumulative percent of genes (y-axis) within each Tn-seq-derived gene set (x-axis) belonging to the functional categories (A) informational, (B) RNA (C) environmental interactions, (D) cellular processes, (E) metabolism and (F) hypothetical is depicted. The proportion of genes contributed by specific sub-KEGG categories is represented by stacked elements within each column. Significant enrichments for KEGG and sub-KEGG categories are denoted with asterisks and white circles, respectively. Gene set abbreviations: hypo-tol. = hypo-tolerant; hyper-tol. = hyper-tolerant; advant. = advantageous. Theoretical KEGG category compositions for Tn-seq gene sets and *p* values were determined using 10,000 Monte Carlo simulations (Methods).

Beyond targeting T+A dinucleotides, transposable mariner elements have a relatively low insertional bias (Table S1 and Figure 2: F11 genome) [27]. Using sequence data from the three input samples exclusively, the number of insertion sequences mapped per 100 bp for all annotated regions was calculated to determine an insertion frequency (Table S2, Methods). As anticipated, we observed that transposon insertion rates within our mutant libraries mostly occurred at a steady rate independent of chromosomal region. An approach akin to traditional genetic footprinting was used to determine the specific insertional tolerance of each gene within the F11 genome [28], [29]. Gene insertion rates that were more than 1 standard deviation below the genome-wide mean were considered hypo-tolerant (<2.1 inserts/100 bp, *n* = 609, Figure 2: hypo-tolerant), those within +/−1 standard deviation were tolerant (2.1 < inserts/100 bp <4.5, *n* = 4,272) and those that were more than 1 standard deviation above the genome-wide mean were hyper-tolerant (>4.5 inserts/100 bp, *n* = 431, Figure 2: hyper-tolerant). Genes assigned to the hypo-tolerant gene set presumably provide ‘essential’ or ‘core’ physiological functions. In contrast, genes within the hyper-tolerant gene set are likely not required during growth in broth culture and/or exhibit certain genetic features that support high rates of transposon insertion. Using the Kyoto Encyclopedia of Genes and Genomes (KEGG) functional hierarchies, the hypo-tolerant gene set, as expected, was found to be significantly enriched for informational functions related to transcription and translation and included various noncoding RNAs (ncRNA) and transfer RNAs (tRNA) (Figure 3A and B). The hyper-tolerant gene set contained a significantly elevated proportion of ribosomal RNAs, ncRNAs and genes annotated as ‘hypothetical’ (Figure 3B and F).

#### Genes required for *in vivo* fitness

To identify genes necessary for *in vivo* fitness, a modified genetic footprinting approach was used (Figure S5). For input-output pairs in each experimental replicate (i.e. insertion mutants present in cognate input and output pools), a normalized occurrence frequency reflecting their abundance was calculated. This approach relies on an ability to count the number of specific mutant variants in corresponding input and output pools. However, strong negative selection within the zebrafish host may have completely purged variants with disruptions in genes critical for fitness. Therefore, we established criteria to determine which mutant variants were likely lost due to selection and not by chance through experimental bottlenecks (Figure S5 and Methods). Missing variants that met these criteria were assigned a value equal to the limit of detection. Within each replicate experiment, we employed a Wilcoxon signed-rank test on the collection of inserts for each gene to determine if the distribution of insert occurrence frequencies between input and output samples was significantly different. This is a conservative approach that requires consistency between insertion variants across a given gene for it to be identified as a fitness determinant. Genes with 10 or more measured inserts, a Wilcoxon *p* value less than 0.05 and an average decrease in variant abundance of at least 2-fold were considered to have a significant influence on *in vivo* fitness (FDR <10%). Of the 970 genes identified across all replicates and niches, only 21 overlapped with the hypo-tolerant gene set, suggesting that the vast majority of genes identified are specifically required by F11 to survive within the zebrafish embryo (Figure 2: *in piscis*, http://pathogenomics.path.utah.edu/F11_TnSeq/). This ‘*in piscis*’ gene set represents ∼18% of the annotated regions within the F11 chromosome and is enriched for genes with functions that fall under the KEGG category ‘environmental interactions’ and the subcategories membrane and iron transport (Figure 3C). Additionally, metabolic functions were also highly represented, encompassing genes involved in glycan biosynthesis and the metabolism of amino acids and carbohydrates (Figure 3E). The proportion of candidate fitness genes that were identified within each of the three mutant libraries is visualized in Figure S6. Genes within the six different *in piscis* gene sets showed only weak, though still in some cases significant, overlap (denoted in Figure S6), suggesting that the screening of multiple libraries was important for a more thorough enumeration of regions that influence *in vivo* fitness. Of note, we speculate that we did not fully reach a level of experimental saturation that matched the mutagenic saturation of our libraries. This observation may indicate a point of consideration for future Tn-seq-based screens using high-throughput surrogate hosts such as zebrafish where initial inoculum doses are relatively small and the stochastic nature of infection can influence the resolution of more subtle phenotypes. However, based on the findings presented in the following sections, we conclude that this perceived experimental noise did not compromise our ability to accurately identify and confirm genes of importance to ExPEC fitness and virulence.

#### Niche-dependent gene requirements

Seventy-six genes (1.4% of total gene content) were found to be necessary for fitness during both localized (PC) and systemic (blood) infections (http://pathogenomics.path.utah.edu/F11_TnSeq/). This ‘multi-niche’ gene set appears to collectively encode functions that directly influence gene expression and membrane architecture, both of which are known to be critical for pathogen fitness. Indeed, a number of the multi-niche genes have already been recognized for their role in bacterial stress resistance, regulation of virulence genes and pathogenesis (Table 1). Transposon inserts within several of the multi-niche genes resulted in some of the most dramatic declines in F11 fitness *in vivo* (Table 1). Notable candidates include *EcF11_3256/bipA*, *EcF11_1255/lrhA* and *EcF11_2271/rfaH* [30], [31], [32]. This gene set is also remarkably enriched for factors that function in glycan biosynthesis, including several genes involved in the production of capsular polysaccharides, which is a well-documented ExPEC-associated virulence trait (Figure 3E) [33], [34].

**Table 1:** Biological significance of Tn-seq-derived candidate genes.

Category	Locus tag	Gene name	*p* value	log_2_ change	Niche	KEGG classification (subcategory)	Literature-based biological significance	Model system
**Informational**	*EcF11_3256*	*bipA*, *typA*	<0.0001	−8.12	blood,PC	Unclassified	Cold stress response [53]	*E. coli* K12 lab strain/*in vitro*
							Co-ordination of pathogenicity island gene expression [30]	EPEC/*in vitro*
							BipA binds ribosome under stress conditions [35]	*Salmonella enterica*/*in vitro*
							Stress adaptation and symbiosis [36]	*Sinorhizobium meliloti*/*in vivo* plant model
	*EcF11_5238*	*hdeD*	0.0128	−4.80	blood,PC	Hypothetical	Acid stress regulon [54]	*E. coli* K12 lab strain/*in vitro*
	*EcF11_1255*	*lrhA*	0.0001	−3.35	blood,PC	Hypothetical	Regulation of virulence genes [31]	EHEC/*in vitro*
							Regulation of motility, lipase activity, toxin expression and virulence [55]	*Xenorhabdus nematophila*/*in vivo* insect model
	*EcF11_2271*	*rfaH*	0.0002	−7.29	blood,PC	Informational (transcription factor)	Global regulator of virulence [32]	UPEC/*in vitro* and *in vivo* mouse model
	*EcF11_4315*	*nhaR*	0.0121	−1.36	blood,PC	Informational (transcription factor)	Regulates genes that facilitate cell-cell adhesion and adhesion to host cells [56]	*Proteus Mirabilis*/*in vivo* mouse model
	*EcF11_0317*	*sirB1*	0.0268	−3.26	blood	Hypothetical	Regulation of the SPI type III secretion system [67]	*Salmonella typhimurium*/*in vitro*
	*EcF11_5302*	*hdfR*	0.0019	−3.44	blood	Hypothetical	Regulator of the *std* fimbrial operon [68]	*Salmonella enterica*/*in vitro*
							Regulates flagellar synthesis [69]	*E. coli* K12 lab strain/*in vitro*
**Membrane architecture**	*EcF11_1390*	*kpsM*	0.0033	−8.11	blood,PC	Environmental interactions (transporter)	Capsular polysaccharide exporter [70]	*E. coli* K1/*in vitro*
							KpsM is predominantly encoded by strains of the *E. coli* B2 phylotype [71]	*E. coli*/*in silico*
							Resistance against phagocyte-mediated killing [33]	ExPEC/tissue culture
	*EcF11_1396*	*kpsS*	0.0229	−3.84	blood,PC	Unclassified	Capsular polysaccharide exporter [72]	*E. coli* K5/*in vitro*
	*EcF11_1397*	*kpsC*	0.0035	−2.57	blood	Unclassified	Capsular polysaccharide exporter [73]	*E. coli* K5/*in vitro*
	*EcF11_1398*	*kpsU*	0.0018	−4.78	blood,PC	Metabolism (lipopolysaccharide biosynthesis)	Capsular polysaccharide exporter [73]	*E. coli* K5/*in vitro*
	*EcF11_1399*	*kpsD*	0.0067	−1.95	blood,PC	Hypothetical	Capsular polysaccharide exporter [74]	*E. coli* K5/*in vitro*
	*EcF11_1400*	*kpsE*	0.0006	−3.40	blood,PC	Metabolism (lipopolysaccharide biosynthesis)	Capsular polysaccharide exporter [57]	*E. coli*
							Capsule production and adhesion/invasion of host cells [75]	*Campylobacter jejuni*/*in vitro* and tissue culture
	*EcF11_1401*	*kpsF*	0.0192	−2.66	blood,PC	Metabolism (lipopolysaccharide biosynthesis)	Capsular polysaccharide exporter [76]	*E. coli* K1/*in vitro*
	*EcF11_0979*	*wzzB*	0.0480	−2.39	blood,PC	Metabolism (lipopolysaccharide biosynthesis)	Regulation of O-antigen chain length [77]	*Salmonella typhimurium* and *Shigella flexner*i/*in vitro*
**Nutrient utilization**	*EcF11_3352*	*manA*	0.0024	−3.20	blood,PC	Metabolism (fructose and mannose metabolism)	Mannose utilization, exopolysaccharide structure, biofilm formation and virulence [78]	*Photorhabdus Luminescens*/*in vitro* and *in vivo* insect model
	*EcF11_2113*	*fitD*	0.0048	−2.87	blood	Environmental interactions (transporter)	Faciliates iron uptake [79]	various *E. coli*/*in vitro*
	*EcF11_4915*	*eutJ*	0.0191	−2.14	blood	Unclassified	Utilization of ethanolamine as a carbon source [80]	*Salmonella typhimurium*/*in vitro*
	*EcF11_4920*	*eutT*	0.0096	−2.49	blood	Metabolism (porphyrin and chlorophyll metabolism)	Utilization of ethanolamine as a carbon and nitrogen source [81]	*Salmonella enterica*/*in vitro*
**Other**	*EcF11_0498*	*tehA*	0.0188	−1.68	blood	Unclassified	Resistance to antiseptics and disinfectants [82]	*E. coli* K12 lab strain/*in vitro*
	*EcF11_2210*	*tieB*, *senB*	0.0002	−2.81	blood	Hypothetical	Entertoxin produced by *E. coli* and *Shigella* [83]	EIEC/*ex vivo* rabbit ileum
	*EcF11_3975*	*ompR*	0.0164	−1.97	blood	Environmental interactions (two-component system)	Osmotic stress resistance and fitness in a mouse urinary tract infection model [84]	UPEC/*in vitro* and *in vivo* mouse model
	*EcF11_2727*	*potD*	0.0063	−1.44	PC	Environmental interactions (transporter)	Polyamine surface protein required for virulence [85]	*Streptococcus pneumoniae*/*in vivo* mouse model

As noted above, the zebrafish PC is more permissive to ExPEC growth than the bloodstream [10], [11]. Corroborating this observation, we found 772 genes to be exclusively required for blood-borne fitness while only 122 genes were exclusive to the PC (http://pathogenomics.path.utah.edu/F11_TnSeq/). This blood-specific gene set was enriched for 7 different KEGG functional categories, whereas the PC-specific gene set was enriched for only 1 (Figure 3, *p*<0.05). Iron transport, the sole category enriched within the PC gene set, was also enriched within the blood set (Figure 3C). Additional categories that were enriched among blood-borne fitness genes covered signal transduction, membrane transport and the metabolism of amino acids, carbohydrates and lipids (Figure 3C and E). These observations indicate that the ability of ExPEC to survive and grow systemically within the zebrafish is, in part, dictated by a functionally diverse set of genes that facilitate the utilization of nutrients that are likely scavenged from the host.

#### Retrospective validation of a Tn-seq-derived fitness determinant

**Figure 4:**
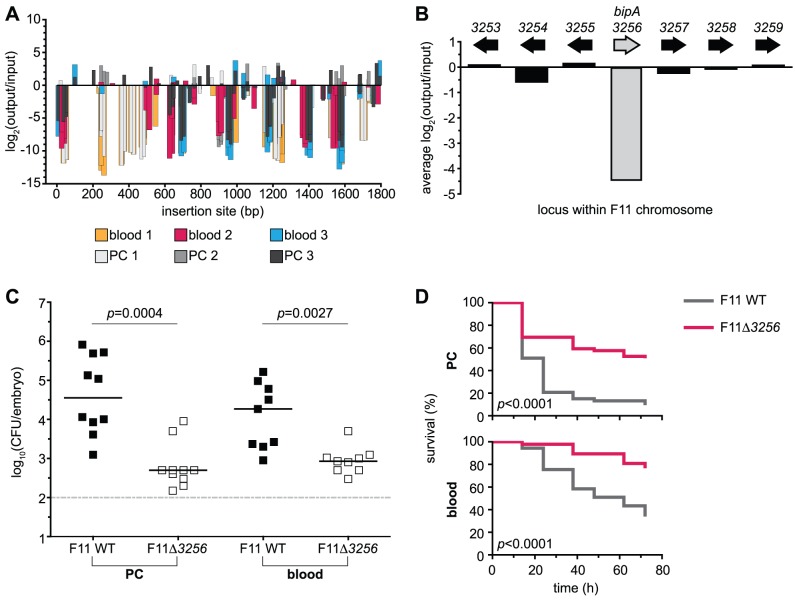
Retrospective deletion of the candidate gene *EcF11_3256/bipA* confirms Tn-seq as a useful tool for the identification of loci required for fitness and virulence. (A) Inserts found within the locus *EcF11_3256/bipA* are plotted with respect to their position of integration (x-axis). Magnitude and direction of bars represent the change in fitness of F11 (y-axis) that correlated with a given insertion event. Color of bars denotes pool of origin. Changes in fitness are presented as log_2_ of the ratio of occurrence frequencies observed between output and input pools (output/input). (B) The average of insert-based fitness changes in (A) is plotted on the x-axis (shaded bar, *bipA*). For comparison, the average alterations in occurrence frequency ratios for proximal genes are also plotted. (C) Equal numbers (1,000–2,000 CFU total) of wild type and F11Δ*3256* were inoculated into the PC (left) or bloodstream (right) of zebrafish embryos. Fish were sacrificed and bacterial loads enumerated ∼20 h post-inoculation by differential plating (*n* = 9 to 10 zebrafish). Dashed line marks the limit of quantification; *p* values were determined using a paired t-test, bars indicate medians. (D) The pericardial cavity (PC, top) and blood (bottom) of 48 hpf embryos were inoculated with approximately 3,000 CFU. Fish were scored for death at 0, 12, 24, 36, 48, 60 and 72 h post-inoculation (hpi). Data are presented as Kaplan-Meier survival plots and *p* values were calculated using a log-rank (Mantel-Cox) test (*n*>45 for each survival curve).

We chose the candidate gene *EcF11_3256*/*bipA*—which has not been previously reported to play a part in the virulence potential of ExPEC—to experimentally confirm the proficiency of our Tn-seq approach. An insertion map of this gene, depicting the reduction in fitness for each variant across all six pools, is presented in Figure 4A. The average changes in fitness for *bipA* and neighboring genes were also examined and indicated that the disruption of only *bipA* correlated with the observed fitness defect (Figure 4B). BipA is conserved across several bacterial phyla and has previously been shown to be a ribosome-associated GTPase that can coordinate the expression of virulence and stress response genes [30], [35], [36]. A targeted deletion of the *EcF11_3256/bipA* allele was generated and the resulting mutant derivative injected into the zebrafish PC and CV at a one-to-one ratio with wild type F11. F11Δ*3256* exhibited a significant reduction in fitness compared to the wild type strain in both environments (Figure 4C). Independent challenges within the PC and bloodstream revealed that F11Δ*3256* also exhibits significantly attenuated killing kinetics compared to wild type (Figure 4D). These results confirm that *EcF11_3256/bipA* is a critical component of F11's genetic repertoire and promotes both the fitness and virulence of this ExPEC strain within distinct host environments. Collectively, the identification of genes with known roles in bacterial pathogenesis (Table 1) and our observations with the Tn-seq-derived *in piscis* candidate gene *EcF11_3256/bipA* validate the Tn-seq protocol described here as a viable approach for detecting fitness and virulence determinants of ExPEC.

#### Disruptions that confer an *in vivo* fitness advantage

We identified 227 genes (∼4% of gene content), that when disrupted conferred a fitness advantage to F11 within the zebrafish host (http://pathogenomics.path.utah.edu/F11_TnSeq/). Twenty of these gene disruptions were advantageous in both the PC and blood, 124 were advantageous only in the PC and 84 conveyed significant advantages in only the blood. KEGG functional categories enriched within the ‘advantageous’ gene set include cell motility, nucleotide metabolism and biosynthesis of vitamins and cofactors (Figure 3D and E). This indicates that motility is likely dispensable for survival within zebrafish and agrees with previous work showing that loss of this trait can provide bacteria with a fitness advantage in some host settings [37]. Other factors encoded within the advantageous gene set may control processes that are easily trans-complemented by neighboring bacterial cells through the production of ‘common goods’ or represent negative regulators of virulence determinants that promote competitive growth [38]. Thirteen of the 227 advantageous genes were hyper-tolerant to transposon insertion, suggesting that the inactivation of these genes may have provided a growth advantage prior to *in piscis* selection (Figure 2: advantageous).

### Trait Enrichment Analysis (TEA) is used to filter Tn-seq-derived candidate genes for putative virulence determinants

To focus the list of Tn-seq-derived candidate genes for further study, we developed a qualitative ranking system using a customized algorithm referred to as **T**rait **E**nrichment **A**nalysis (TEA). The underlying premise of TEA was inspired by molecular Koch's postulates, which are a widely acknowledged set of guidelines used by investigators to critically assess the contributions made by certain genetic elements to the virulent nature of pathogenic organisms [39]. In particular, TEA draws on the first axiom of these postulates, which states that, “*The phenotype or property under investigation should be associated with pathogenic members of a genus or pathogenic strains of a species.*” [40]. Thus, the primary goal of TEA is to identify correlations between bacterial genes and bacterial traits (i.e. genotype-phenotype relationships).

**Figure 5:**
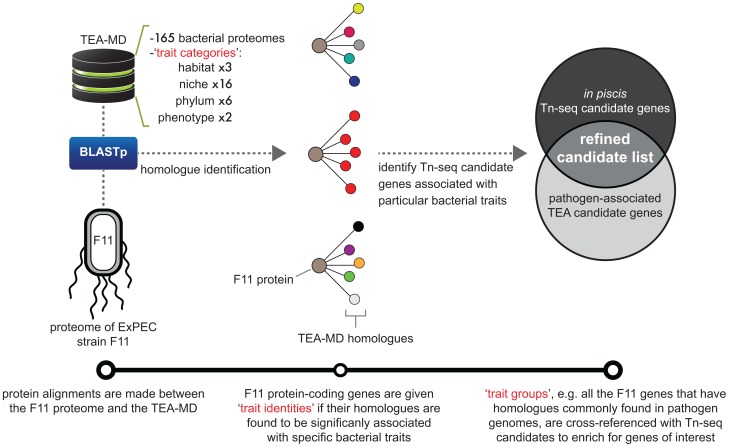
Diagrammatic workflow of ‘Trait Enrichment Analysis’ (TEA). This visualization depicts the TEA method and describes the relationships between various data types. Bottom track: linear representation of steps followed in the execution of TEA. Protein alignments are made between F11 proteins and the TEA-MD to identify homologues. Microbes within the TEA-MD are annotated with four separate traits contained within the trait categories: habitat (1 of 3), niche (1 of 16), phylum (1 of 6) and phenotype (1 of 2). The composition of traits associated with homologue sets for each F11 protein is assessed for enriched traits in order to assign trait identities. Trait groups, which are F11 genes that share a particular trait identity, are used to organize Tn-seq-derived candidate genes. TEA-MD = TEA-metaproteome database; BLASTp = Basic Local Alignment Search Tool used to align protein sequences.

TEA was carried out as outlined in Figure 5. Briefly, protein sequences were acquired from an ecologically diverse, manually curated collection of 165 bacteria representing six phyla and assembled to form the TEA metaproteome database (TEA-MD, Table S3). Each bacterial strain was annotated with ‘trait categories’ defining habitat, niche of isolation, taxonomic lineage and phenotype (i.e. pathogen or non-pathogen). Homologues were retrieved for each of the 5,146 protein-coding genes contained within the F11 chromosome using the Basic Local Alignment Search Tool: BLASTp (Methods) [41]. Because proteins in the TEA-MD are linked to a set of traits defined by their source bacterium, homologue collections associated with F11 proteins can be appraised for the presence of a universal characteristic in a similar fashion to traditional gene ontology and KEGG enrichment analyses. As a result, hypotheses can then be generated regarding a protein's evolutionary origins (i.e. horizontal vs. vertical inheritance or homoplasy) and possible influence on bacterial physiology or behavior based on its combination of trait associations. A complete description of the TEA-MD, including the number of organisms and proteins comprising each trait category and the relative proportions of phyla compared to sequenced isolates in GenBank, is provided as supporting information (Figures S7 and S8).

#### Pathogen-associated genes within Tn-seq gene sets

TEA, as it was employed here, aimed to assign ‘trait identities’ to F11 genes, with particular attention being paid to genes that are common to known pathogens and therefore, putative virulence determinants (Figure 5). It was found that approximately 22% of the F11 genome is made up of genes with pathogenic identity—1,166 F11 genes are significantly more associated with pathogen genomes (Methods). Considering Tn-seq-derived candidate genes, it was anticipated that the *in piscis* gene set be enriched for these types of genes. Indeed, a significant portion of the 970 *in piscis* genes had pathogenic identity (246 genes, 25% of gene set, *p* = 0.009) (Table S4 and http://pathogenomics.path.utah.edu/F11_TnSeq/). The enrichment of pathogen-associated genes within the *in piscis* gene set was largely attributed to the composition of both the PC and blood gene sets, which also had a significant number of genes with pathogenic identity as determined by TEA (PC: 39 genes, ∼32% of gene set, *p* = 0.008; blood: 192 genes, ∼25% of gene set, *p* = 0.0485). Interestingly, the multi-niche gene set was not enriched for pathogen-associated genes (15 genes, ∼20% of gene set, *p* = 0.786).

We conclude from these data that genes required for F11 fitness in multiple niches are likely to encode more basic ‘core’ type functions that may enable both pathogenic and non-pathogenic strains alike to better deal with general stresses encountered within varied host environments, but not necessarily in broth culture. In contrast, the niche-specific genes, which are more likely to be pathogen-associated, may endow bacteria like F11 with salient, pathoadaptive traits that are perhaps contextually regulated. Along these lines, we unexpectedly found that the advantageous gene set was also enriched for pathogen-associated genes (Table S4, 69 genes, ∼30% of gene set, *p* = 0.004). We hypothesize that the disruption of these particular genes during the Tn-seq selection screen alleviated fitness costs associated with their maintenance and/or expression within the zebrafish host. Future investigation of the advantageous gene set may illuminate evolutionary tradeoffs that are incurred by the acquisition and retention of these loci by pathogens as they traverse different host-associated environments.

#### Examination of Tn-seq candidates filtered by TEA

**Figure 6:**
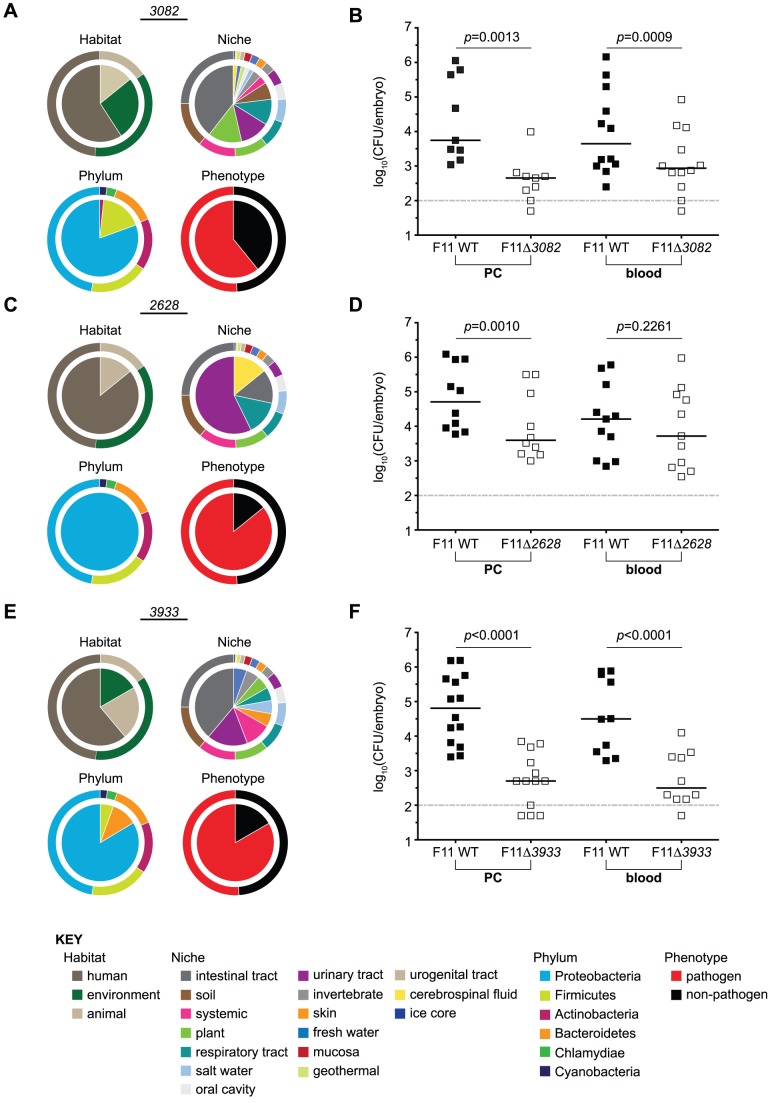
Targeted deletion of candidate genes with pathogenic identity attenuates the fitness of F11 within zebrafish. The composition of traits (bottom KEY) represented among bacteria encoding homologues of (A) *EcF11_3082*, (C) *EcF11_2628*, or (E) *EcF11_3933* are presented for the trait categories habitat of isolation, niche of isolation, phylum and phenotype. For reference, segmented outer rings bordering each pie graph depict the composition of traits of all genes contained within the TEA-MD. Equal numbers (1,000–2,000 CFU total) of wild type and (B) F11Δ*3082*, (D) F11Δ*2628*, or (F) F11Δ*3933* were inoculated into the PC (left) or bloodstream (right) of embryos. Fish were sacrificed and bacterial counts determined ∼20 h post-inoculation by differential plating (*n* = 9 to 14 zebrafish). Dashed line marks the limit of quantification; *p* values were determined using a paired t-test, bars indicate medians.

To investigate the effectiveness of TEA to help identify previously uncharacterized genes important for ExPEC fitness *in vivo*, we selected 3 from the subset of 246 pathogen-associated Tn-seq-derived *in piscis* genes for closer examination. *EcF11_3082*, *EcF11_2628* and *EcF11_3933* were individually deleted from the F11 chromosome and tested in competitive infection assays against wild type F11 within the zebrafish PC and blood. These genes were chosen based on their performance in the initial Tn-seq selection screen and their association with known pathogens, as determined by TEA. Trait identities for each gene are illustrated in Figure 6 along with the results from competitive infection assays.

The *EcF11_3082* gene product is homologous to the transcriptional repressor MprA/EmrR. In *E. coli* lab strains, MprA/EmrR has been shown to negatively regulate biosynthesis of the microcin B17 antibiotic and a multidrug efflux pump encoded by *emrA and emrB* [42], [43]. Fifty-six strains within the TEA-MD harbor an *EcF11_3082*-like gene, 34 (61%) of which are pathogens (Figure 6A, significance of pathogenic identity: *p* = 0.034). Tn-seq indicated that *EcF11_3082* promotes the fitness of F11 in both the PC and bloodstream in all three replicates. Average fitness deficits observed through Tn-seq for *EcF11_3082* insertion mutants ranged from 5 to 21-fold. This was confirmed by one-to-one competitive assays in which survival of the *EcF11_3082* deletion mutant was markedly diminished relative to wild type F11 following inoculation of the PC and blood (Figure 6B).

Transposon insertions into the *EcF11_2628* locus decreased the fitness of F11 within the blood by an average of 15-fold, as assessed by Tn-seq. The TEA-MD contains 7 homologues of *EcF11_2628*, 6 of which belong to ExPEC strains that were isolated from a variety of host-associated niches (Figure 6C, significance of pathogenic identity: *p* = 0.035). *EcF11_2628* encodes a hypothetical protein that is predicted by PHYRE (Protein Homology/analogY Recognition Engine) to function as a toxic extracellular metalloendopeptidase [44]. A structurally similar toxin, AsaP1, was recently described as a critical virulence factor encoded by the natural fish pathogen *Aeromonas salmonicida subsp. Achromogenes* [45]. Deletion of this gene from F11 resulted in decreased fitness within the PC and surprisingly, had no discernible effects in the bloodstream during one-to-one competition assays with the wild type strain (Figure 6D). These results run counter to those obtained with Tn-seq, where disruption of *EcF11_2628* reduced the fitness of F11 within the blood. This discrepancy may be attributable to the fact that Tn-seq is in effect a one-to-3,000 competitive assay among transposon insertion mutants within each fish, whereas in our follow-up assays specific gene knockout mutants are tested in one-to-one competition with wild type. Within these different experiments, individual mutants may be subject to very different pressures and frequency-dependent selection events.

Tn-seq results indicated that disruption of a third pathogen-associated gene, *EcF11_3933*, attenuates the fitness of F11 about 4-fold within the PC. Fifteen of the 18 EcF11_3933 homologues (83%) found in the TEA-MD are encoded by known pathogens, including multiple ExPEC strains, *E. coli* O157:H7, *Neisseria gonorrhoeae*, *Vibrio cholera*, *Haemophilus influenza*, *Photorhabdus luminescens* and *Erwinia amylovora* (Figure 6E, significance of pathogenic identity: *p* = 0.001). EcF11_3933 is uncharacterized and annotated as a homologue of the broadly conserved DNA protecting protein DprA. A related but distinct protein, DprA/Smf, has been suggested to confer competence for the uptake of foreign DNA by various bacterial species [46], [47], though it is presumed to function differently in lab strains of *E. coli* [48]. In competitive assays, deletion of *EcF11_3933* rendered F11 significantly less fit than the wild type strain in both the PC and bloodstream of zebrafish (Figure 6F).

### Tn-seq and TEA-derived *in piscis* candidate genes contribute to pathogen fitness within mouse models of bacteremia and urinary tract infection

**Figure 7:**
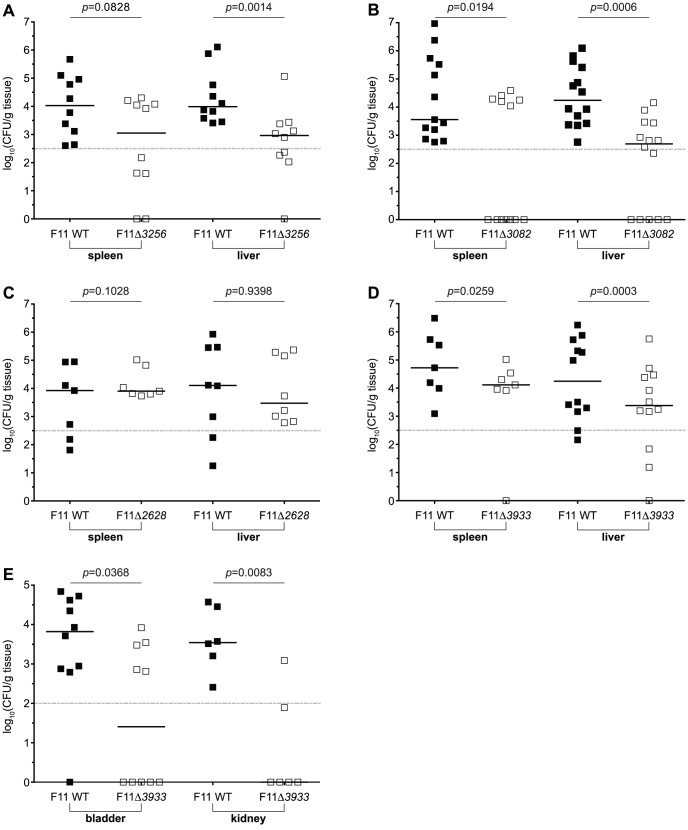
Tn-seq and TEA-derived candidate genes promote the fitness of F11 within murine models of bacteremia and urinary tract infection. Equal numbers (10^8^ total CFU) of wild type F11 and (A) F11Δ*3256*, (B) F11Δ*3082*, (C) F11Δ*2628*, or (D) F11Δ*3933*, were subcutaneously injected into the nape of the neck of adult female Swiss Webster mice. Approximately 12 to 15 h later, bacterial titers within the spleen (left) and liver (right) were determined by differential plating. (E) Equal numbers (10^7^ total CFU) of wild type F11 and F11Δ*3933* were transurethrally inoculated into the urinary tract of adult female CBA/J mice. Bacterial titers present in the bladder (left) and kidneys (right) were determined 3 d post-inoculation by differential plating. Dashed line marks the limit of quantification. *n* = 7 to 14 mice; *p* values were determined using a paired t-test, bars indicate medians.

To extend our experimental and sequence-based observations with the candidate genes *EcF11_3256*/*bipA*, *EcF11_3082*, *EcF11_2628* and *EcF11_3933*, we employed a more traditional host organism for investigating ExPEC pathogenicity—the laboratory mouse [49], [50]. Evaluating Tn-seq and TEA-derived *in piscis* candidate genes using a murine model provided an opportunity to further establish the biological relevance of our multilayered approach. Murine models of bacteremia are an established means to assess the general capacity of ExPEC isolates to replicate and cause disease within a host [50], [51], [52]. One-to-one mixtures of wild type F11 and each mutant strain (10^8^ total CFU) were subcutaneously injected into adult female Swiss Webster mice. Approximately 12 to 15 h later, bacterial titers within the spleen and liver were determined (Figure 7). Mutants lacking *EcF11_3256*/*bipA* (Figure 7A), *EcF11_3082* (Figure 7B) and *EcF11_3933* (Figure 7D) were significantly outcompeted by the wild type F11 parent strain. However, F11Δ*3256* exhibited a more modest defect in the spleen (*p* = 0.0828). In contrast, there was no observable competitive difference between wild type and F11Δ*2628* in either organ (Figure 7C).

Of note, we found that F11Δ*3256* displayed a cold sensitivity phenotype when grown at 20°C in broth culture, which is consistent with reports on BipA function in other bacteria (Figure S9A) [53]. Therefore, we were initially concerned that the growth rate of this mutant at 28.5°C—the temperature used to raise zebrafish embryos—was compromising our ability to distinguish its temperature and host-dependent phenotypes. However, F11Δ*3256*, like the other mutants, grew similar to the wild type strain at both at 28.5°C and 37°C (Figure S9). We conclude that the attenuated fitness phenotypes observed with F11Δ*3256* in competitive assays in both zebrafish and mice are independent of temperature effects.

We next tested the fitness contributions made by each of the four candidate genes above within the murine urinary tract, which is another commonly used model system for understanding ExPEC pathogenicity. Three days after inoculation of one-to-one mixtures of wild type and mutant derivatives (10^7^ total CFU) into the urinary tract of adult female CBA/J mice, kidneys and bladders were harvested and bacterial titers determined. Of the four genes assayed, only *EcF11_3933* was critical for the competitive fitness of F11 within the bladder and kidneys (Figure 7E).

Our ability to identify candidate genes necessary for ExPEC fitness within mammalian host niches through initial prescreening in embryonic zebrafish highlight the utility of this teleost host to model overlapping aspects of ExPEC biology. By comparing the specific host and niche-specific phenotypes of various ExPEC isolates and mutant strains in futures investigations, we will be able to gain a deeper understanding of the host-pathogen interactions engaged by ExPEC.

## Discussion

### Application of Tn-seq to investigate the multigenic basis of ExPEC pathogenicity

The genetic and phenotypic plasticity of ExPEC isolates, which is in part due to horizontal transfer of pathoadpative accessory genes, presumably enables these pathogens to traverse a wide range of environmental and host-associated niches [7], [8], [10], [11]. It will be important to decipher how ExPEC employ their genetic repertoires so that more specific and efficacious therapeutic strategies can be developed. Moreover, knowledge of key events that occur during the evolutionary assembly of pathogen genomes will likely be gleaned as genotype-phenotype relationships are mapped. To this end, we functionally dissected the contents of an ExPEC reference genome using the recently described transposon-based screening technique Tn-seq (Figure 1) [14], [15]. With mutant pools derived from the ExPEC reference isolate F11 and multi-niche zebrafish infection models, we found that ∼18% of the F11 genome significantly affected bacterial fitness under the pathogenic conditions tested. These fitness determinants can be grouped based on the niches where they were required, which in some instances correlated with particular functions like iron transport and amino acid metabolism (Figure 3). Gene set and gene-based contributions to fitness can be explored through an online and curated data viewer (http://pathogenomics.path.utah.edu/F11_TnSeq/). Going forward, it is our intent that this work will expedite future comparative and functional pathogenomic analyses focused on elucidating the genetic basis of pathogen behavior and evolution.

Seventy-six genes (1.4% of genome content) were found to significantly influence the fitness of F11 in both the PC and blood of zebrafish. We suggest that these multi-niche genes largely provide core functions that are critical, but not necessarily specific, to pathogen physiology. It was not surprising that several of these genes were recognizable and annotated as being involved in the regulation of gene expression and membrane architecture—two functions that are important to pathogens and non-pathogens alike (Table 1). In addition to the translational regulator EcF11_3256/BipA, which we validated in follow up experiments using a targeted deletion mutant and multiple *in vivo* host model systems (Figure 4 and 7A), several other informational genes, including *hdeD*, *lrhA*, *rfaH* and *nhaR*, were also identified by our Tn-seq screens as being important to the fitness of F11 within multiple niches. These genes have been shown to affect acid stress resistance, toxin expression, adhesion to host tissues and virulence gene regulation in a diverse array of pathogens, including enterohemorrhagic *E. coli* (O157:H7), *Xenorhabdus nematophila*, *Proteus mirabilis* and a sub-group of ExPEC isolates like F11 known as uropathogenic *E. coli* (UPEC) (Table 1) [31], [32], [54], [55], [56]. Additionally, genes responsible for the production of capsule—which is a well-established virulence feature that serves as a protective polysaccharide barrier surrounding the bacterial cell wall—were also important in both the PC and blood (Table 1) [57]. Further identification and, importantly, functional characterization of multi-niche genes will lead to a deeper understanding of the general fitness and virulence gene networks employed by ExPEC and other virulent bacteria within varying host environments.

Aside from multi-niche genes, we observed a striking numerical imbalance of genes that are exclusively required for either blood-borne or tissue-localized PC fitness (772 and 122 genes, respectively). The large number of genes and variety of enriched KEGG functional categories within the blood-specific gene set corroborated previous experimental observations that indicated the blood of embryonic zebrafish is a hostile environment and highly restrictive to bacterial growth [10], [11]. Specifically, genes involved in nutrient acquisition and utilization made up a significant portion of the blood-specific genes (Figure 3). These results provide evidence that during systemic infections nutrients are more limiting than during localized infection of the PC, requiring ExPEC to employ a greater portion of their genetic repertoire to maintain fitness in the blood. However, additional factors could also account for the discrepancy between the PC and blood-specific gene sets, including instances of trans-complementation. Once injected into the PC, bacteria remain confined until the embryo succumbs to the infection [10]. Because bacteria stay in close proximity to one another within the PC, the chances for trans-complementation and the sharing of common goods are high and may result in relaxed selection. A recent study demonstrated that extensive trans-complementation can occur within mixed mutant populations of *Yersinia pestis* in a mouse model of pneumonic plague [58]. However, the authors noted that this appears to be a unique characteristic of *Y. pestis*, as other pathogens with tropism for lung tissue do not exhibit such a high degree of wild type-to-mutant rescue. In our assays, trans-complementation effects within the PC were apparently limited as we were able to identify genes required for ExPEC survival within this niche.

Ultimately, this initial genome-wide analysis of the components necessary for ExPEC fitness within pathogenic environments garnered two key insights: (i) the number of genes that influence fitness in multiple niches is relatively small and (ii) there are distinct subsets of genes employed by ExPEC within different host environments. This suggests that *do-it-all* genes do not necessarily dictate the generalist and plastic nature of ExPEC. Instead, ExPEC may evolutionarily maintain sets of context-specific genes that can be employed, possibly in different combinations, to respond in a highly specific-manner to a variety of conditions. This characteristic likely contributes to the seemingly redundant composition and versatility of contemporary ExPEC genomes. Further functional dissection of ExPEC genomes by Tn-seq-based approaches will help clarify which genes underlie the salient traits of this lineage and inform the design of broad-spectrum therapies that target only ExPEC while leaving beneficial or benign *E. coli* strains within the normal microbiota unperturbed.

### Refinement of candidate gene sets by TEA

A longstanding challenge associated with unbiased, high-throughput functional genomics techniques like Tn-seq has been the need to develop methods that provide an effective transition to focused follow-up studies. Much work has been done to quantitatively filter datasets using sophisticated determinations of statistical significance and false discovery control procedures. Analysis of the molecular pathways represented within functional genomics gene sets has also been addressed extensively. However, less attention has been given to systematic methods that exploit the qualitative features of individual genes and how such considerations can streamline candidate gene vetting. To address this issue, we developed a **T**rait **E**nrichment **A**nalysis (TEA) tool to organize Tn-seq-derived candidate genes in a biologically meaningful way (Figure 5). With a manually curated set of 165 microbial proteomes, TEA is able to assign meta-features to F11 genes based on their association with specific bacterial traits (i.e. habitat, niche of isolation, phylum and phenotype). In this way, TEA provides a tractable method to categorize Tn-seq candidate genes into a more focused subset—something not easily accomplished using larger microbial sequence databases like GenBank and BioCyc or functional annotation platforms such as DAVID. TEA uncovered several genes with previously unappreciated roles in ExPEC pathogenicity, thus creating new opportunities to further understand the genetic underpinnings of this lineage.

For the purposes of our investigation, we used TEA to identify F11 genes with homologues that are more often associated with the genomes of known pathogens. We describe such genes as having ‘pathogenic identity’ and reason that, because of their affiliation with pathogens, they are more likely to contribute to pathogenic behavior—a principal tenet of molecular Koch's postulates [39], [40]. In considering the overlap between Tn-seq-derived *in piscis* genes and the TEA-derived gene set made up of F11 genes with pathogenic identity, we were able to identify 246 genes that potentially influence the *in vivo* fitness capacity of F11 specifically. We selected three of these pathogen-associated candidate genes for closer inspection.

The candidate gene *EcF11_3082* was identified by Tn-seq and confirmed during one-to-one co-challenge experiments to be important for the fitness of F11 within the blood and PC of zebrafish and spleen and liver of mice (Figure 6B and 7B). This gene appears to be relatively widespread within the TEA-MD, being found in 56 genomes, 61% of which are classified as pathogens. The range of bacteria encoding homologues of EcF11_3082 includes isolates from 12 (out of 16) different niches and 3 (out of 6) phyla represented in the TEA-MD (Figure 6A). These observations suggest that *EcF11_3082* may play a broad role in bacterial fitness within a variety of pathogenic and non-pathogenic contexts. *EcF11_3082* is predicted to encode an MprA/EmrR-like transcriptional repressor, which was shown to regulate the expression of a downstream multidrug efflux pump encoded by *emrA* and *emrB*. Although our Tn-seq selection screen and co-challenge experiments identified EcF11_3082 as a fitness determinant, disruption of the *emrA* and *emrB* genes present in F11 did not appreciably affect fitness within the zebrafish host, as determined by Tn-seq. This suggests that either disruption of *EcF11_3082* led to aberrant and deleterious expression of *emrAB* that resulted in attenuation of the *EcF11_3082* mutant, or the particular MprA/EmrR allele harbored by F11 influences other aspects of bacterial physiology not yet defined. Further investigation is required to determine the exact influence of *EcF11_3082* on pathogenicity.

*EcF11_2628*, which encodes a ‘conserved hypothetical protein’ that has some homology to a secreted metalloendopeptidase toxin, was also identified by Tn-seq and confirmed as an important fitness determinant using a targeted knockout strain (Figure 6D). However, we were unable to ascertain if this gene is required during colonization of a mammalian host. The F11Δ*2628* mutant variant did not exhibit any statistically significant decline in competitive fitness during systemic or urinary tract infection of mice (Figure 7C and data not shown). Further investigation is required to determine the physiological context in which this putative toxin promotes ExPEC fitness. Nonetheless, homologues of *EcF11_2628* were found in only 7 proteobacterial genomes within the TEA-MD (Figure 6C and D). Of these, 5 are ExPEC strains that were isolated from human patients with either meningitis (strain S88) or UTI (strains CFT073, UTI89, 536 and UMN026). Another ExPEC strain (APEC01) harboring an *EcF11_2628* homologue came from a case of avian sepsis that occurred subsequent to respiratory tract infection. The sole non-pathogen that carried an *EcF11_2628* homologue was ED1a, a commensal isolate from the human gut that is classified as belonging to the B2 phylogenetic group—the *E. coli* lineage that encompasses the vast majority of human ExPEC strains [7]. This observation is intriguing because the *EcF11_2628* allele carried by ED1a could represent either a genetic vestige from a pathogenic past or a new acquisition that is edging ED1a down an evolutionary path towards a more ExPEC-like countenance. Elucidating the function of this hypothetical gene should help explain its seemingly restricted distribution within a phylogenetically narrow range of bacteria.

The final pathogen-associated gene that we examined, *EcF11_3933*, encodes a variant of the DNA protection protein DprA [46], [47]. However, this particular *dprA* allele is distinct from those previously characterized in other bacteria. Within the TEA-MD, 83% of the *EcF11_3933* homologues were found in the genomes of known pathogens. Interestingly, pathogens harboring an *EcF11_3933*-like gene included ExPEC isolates as well as other members of Proteobacteria and species within the phyla Bacteroidetes and Firmicutes (Figure 6E). In our Tn-seq selection screens, *EcF11_3933* was initially identified because it appeared to affect fitness within the PC, but subsequent analyses revealed that *EcF11_3933* could also promote pathogen fitness within the bloodstream of both zebrafish and mice (Figure 6F and 7D). Indeed, there were indications that *EcF11_3933* contributed to blood-borne fitness, but it was filtered out of the final Tn-seq-derived blood gene set because it did not meet all of the predetermined cutoffs, though only by a thin margin. These results identify limits to Tn-seq as it is applied here, but also highlight the value of using multiple infection models to discover and assess the roles of fitness and virulence determinants.

Additional work using a murine UTI infection model demonstrated that *EcF11_3933* is also important for competitive fitness within the murine bladder and kidneys (Figure 7E). This is in contrast to *EcF11_3256*, *EcF11_3082* and *EcF11_2628*, which we found to be dispensable for colonization of the urinary tract (data not shown). Interestingly, alleles of *c4222*, which is a homologue *of EcF11_3933* harbored by the UPEC strain CFT073, were shown by microarray to be transcriptionally induced within a panel of UPEC isolates during active infection of the human urinary tract [59]. Together, previous observations and those presented here indicate that *EcF11_3933* is of broad importance to the pathogenic potential of F11 and other ExPEC isolates within a variety host-associated niches. Moreover, the distribution of *EcF11_3933*-like genes among a diverse array of pathogens (e.g. ExPEC, the enteric pathogen *E. coli* O157:H7, *Neisseria gonorrhoeae*, *Vibrio cholera*, *Haemophilus influenza*, *Photorhabdus luminescens* and *Erwinia amylovora*), which can colonize and cause disease within a broad range of animal, insect and plant hosts, suggests that *EcF11_3933* also represents a multi-lineage virulence determinant. Further analysis of *EcF11_3933* and its pathogen-associated homologues may prove useful in defining common virulence strategies employed by ExPEC as well as other more distantly related bacterial species.

### Concluding remarks

Cumulatively, this investigation demonstrates that the application of Tn-seq, coupled with TEA, provides an efficient means to identify novel or previously unappreciated fitness and virulence determinants. The utility of TEA could easily be enhanced through expansion of the TEA-MD by, for example, the addition of metadata obtained from other host-microbe experimental systems. TEA serves as an accelerated annotation layer for bridging high-throughput sequencing experiments and functional characterization. In moving forward, it will be important to continue the careful curation of pertinent biological information associated with the rapidly expanding and often redundant and ill-defined collections of microbial genomics data so that meaningful connections can be mined. Ultimately, the use of Tn-seq and TEA with an expanded number of bacterial isolates and host systems may provide a detailed map of the gene families and signaling cascades employed by ExPEC and other pathogens to colonize host-associated environments.

## Methods

### Ethics statement

Animals used in this study were handled in accordance with University of Utah approved IACUC protocols that followed the standard guidelines described at www.zfin.org and in the Guide for the Care and Use of Laboratory Animals, 8th Edition.

### Bacterial strains and plasmids

All bacterial strains and plasmids used in this study are listed in Table S5. Unless specified otherwise, bacteria were cultured statically at 37°C for 24 h in 20 ml of a defined M9 minimal medium (6 g/L Na_2_HPO_4_, 3 g/L KH_2_PO_4_, 1 g/L NH_4_Cl, 0.5 g/L NaCl, 1 mM MgSO_4_, 0.1 mM CaCl_2_, 0.1% glucose, 0.0025% nicotinic acid, 0.2% casein amino acids and 16.5 mg/ml thiamine in H_2_O) prior to injection into zebrafish embryos or mice. Antibiotics (kanamycin or ampicillin) were added to the growth medium when necessary to maintain recombinant plasmids or select for mutants.

Targeted gene knockouts of Tn-seq-derived candidate genes were generated in ExPEC strain F11 using the lambda Red-mediated linear transformation system [60], [61]. Briefly, a kanamycin resistance gene was amplified using polymerase chain reaction (PCR) from the pKD4 plasmid with 40-base pair overhangs specific to the 5′ and 3′ ends of each targeted locus. PCR products were introduced via electroporation into F11 carrying pKM208, which encodes an IPTG (isopropyl-β-D-thiogalactopyranoside)-inducible lambda red recombinase. Knockouts were confirmed by PCR. Primer sets used are listed in Table S6.

Retrofitting of the previously constructed pSAM_Bt vector to be used for the transposon mutagenesis of ExPEC isolate F11 was accomplished using source vectors and primers as listed in Tables S5 and S6, respectively. The P*_lac_* promoter from pGFP-Mut3.1 (Clonetech), along with its associated ribosome binding sequence, was amplified via PCR. Engineered 5′ BamHI and 3′ NdeI restriction sites were used to sub-clone the resulting fragment into a BamHI/NdeI (New England Biolabs) double digested pSAM_Bt vector upstream of the *himar*1C9 transposase gene. The kanamycin resistance gene from pKD4 was amplified and ligated using the restriction sites MfeI and XbaI, replacing the erythromycin resistance gene *ermG* in pSAM_Bt. The resulting transposon mutagenesis vector, pSAM-Ec, was stored and propagated in the *pir* ^+^ *E. coli* strain EcS17.

### Zebrafish embryos

*AB wild-type zebrafish embryos were collected from a laboratory-breeding colony that was maintained on a 14 h/10 h light/dark cycle. Embryos were grown at 28.5°C in E3 medium (5 mM NaCl, 0.17 mM KCl, 0.4 mM CaCl_2_, 0.16 mM MgSO_4_) containing 0.000016% methylene blue as an anti-fungal agent.

### Infection of zebrafish

One ml from a 24-h bacterial culture of each transposon mutant library or isogenic bacterial strain was pelleted, washed once with 1 ml sterile PBS (Hyclone) and re-suspended in approximately 1 ml PBS to obtain appropriate bacterial densities for microinjection. Prior to injection, 48 h post-fertilization (hpf) embryos were manually dechorionated, briefly anesthetized using 0.77 mM ethyl 3-aminobenzoate methanesulfonate salt (tricaine) (Sigma-Aldrich) and embedded in 0.8% low-melt agarose (MO BIO Laboratories) without tricaine. Approximately 1 nl of bacteria was injected directly into the pericardial cavity (PC) or the blood via the circulation valley (CV) located ventral to the yolk sac using a YOU-1 micromanipulator (Narishige), a Narishige IM-200 microinjector and a JUN-AIR model 3-compressor setup. For each experiment, average colony forming units (CFU) introduced per injection were determined by adding 10 nl of the inoculum into 1 ml 0.7% NaCl that was then serially diluted and plated on Luria-Bertani (LB) agar plates. For co-challenge experiments, input doses were plated on LB agar +/− kanamycin (50 µg/mL) to determine relative numbers of the wild type and mutant strains present. After injection, embryos were carefully extracted from agar and placed in either a 10 cm petri dish containing E3 medium for transposon screening or, individually into wells of a 96-well microtiter plate (Nunc) containing E3 medium for co-challenge experiments. The E3 medium used during this incubation period lacked both tricaine and methylene blue.

To harvest DNA from embryos infected with transposon mutant libraries at the end of the selection period, all infected fish were collected (both alive and dead) in a 1.6 ml tube, being careful to remove as much E3 medium as possible by gently pulsing the tube in a microfuge to facilitate separation. Embryos were suspended in 500 µl of 0.5% Triton X-100 in PBS and homogenized using a mechanical PRO 250 homogenizer (PRO Scientific). Homogenates were then centrifuged at 18,000 g for 5 minutes to sediment bacteria away from host cellular components released during Triton X-100-mediated lysis. The resulting pellet, which appears black because of remaining zebrafish debris, served as the starting material for DNA extraction using the Wizard Genomic DNA Purification Kit (Promega) as per the manufacturer's protocol with a brief modification: the duration of the initial lysis step was extended as needed to dissolve the tough pellet.

To quantify bacterial numbers at the completion of co-challenge experiments, embryos were homogenized at the indicated time points in 500 µL PBS containing 0.5% Triton X-100 using a mechanical PRO 250 homogenizer. Homogenates were then serially diluted and plated on LB agar +/− kanamycin (50 µg/mL) to determine relative numbers of wild type and mutant bacteria. For co-challenge assays, only embryos where the total bacterial CFU recovered was equal to or greater than the limit of quantification (i.e. at least 20 CFU counted on the lowest serial dilution, which corresponds to 100 CFU per embryo) are reported. For lethality assays, fish were inspected for death at the indicated time points over a 72 h period. Death is defined here as the complete absence of heart rhythm and blood flow. Survival graphs depict total pooled results from at least 3 independent experiments in which groups of approximately 15 to 20 embryos were injected at a time.

### Mouse infections

For bacteremic co-challenge experiments, seven to eight-week old female Swiss Webster mice (Charles River) were anesthetized using isoflurane inhalation and injected subcutaneously via the nape of the neck with 200 µl of a 1∶1 wild type to mutant bacterial suspension containing a total of 10^8^ bacteria in sterile PBS. Spleens and livers recovered 12 to 15 h later were weighed and homogenized in 500 µl sterile PBS. Homogenization was done with a Bullet Blender Storm 24 (Next Advance) using three SSB32 3.2 mm stainless steel beads for 5 minutes on power level 6. Homogenates were serially diluted and plated on LB agar +/− kanamycin (50 µg/mL) to determine the number of both wild type and mutant bacteria. Bacteremic co-challenge experiments were repeated at least twice. For these assays, only organs where the total bacterial CFU recovered was equal to or greater than the limit of quantification (i.e. at least 20 CFU counted on the lowest serial dilution, which corresponds to approximately 200 to 600 CFU per g organ) are reported.

For co-challenge experiments within the murine urinary tract infection, seven- to nine-week old female CBA/J mice (Jackson Labs) mice were anesthetized using isoflurane inhalation and inoculated via transurethral catheterization with 50 µl of a 1∶1 wild type to mutant bacterial suspension containing a total of 10^7^ bacteria suspended in PBS. Bladders and kidneys were recovered 3 days later and each was weighed and homogenized in 1 ml containing 0.025% Triton X-100. Homogenates were serially diluted and plated on LB agar +/− kanamycin (50 µg/mL) to determine number of both wild type and mutant bacteria.

### Statistical analysis of zebrafish and mouse infections

For co-challenge experiments, numbers of wild type and mutant bacteria present in the inoculum and recovered from host tissues were enumerated by differential plating on selective media as described above. To determine if bacterial counts differed between wild type and mutant-infected animals, a paired t-test was performed on log_10_-transformed values. Graphs and statistics were generated using GraphPad Prism 5.

### Transposon library generation

#### Transposon mutagenesis

A detailed protocol for the transposon mutagenesis of ExPEC strain F11 using the *E. coli* donor strain EcS17 carrying pSAM-Ec and the methods to verify and sequence mutant libraries is provided in Protocol S1. Briefly, EcS17/pSAM-Ec and F11 were mixed 2∶1 (donor∶recipient), deposited onto nitrocellulose 0.45 µm filter discs (Millipore) and incubated for 5 h on agar plates at 37°C. Because the P*_lac_* promoter allows for constitutive low-level expression of the *himar*1C9 transposase, no induction was required. Bacteria from individual mating discs were recovered in 2 ml of 1× M9 salts by vortexing. A 100 µl aliquot was serially diluted and plated on selective agar plates to determine mutagenesis frequency. The remaining 1.9 ml of mating mixture was added to 20 ml of selective media and allowed to grow shaking at 37°C until an optical density at 600 nm (OD_600_) of ∼0.5 was reached. One ml aliquots from individual matings were then store at −80°C until used in selection screening. In the event a mutant library did not contain a satisfactory number of mutants, for example, <50,000 mutants, frozen axillary stocks were briefly thawed, combined and refrozen.

### Bioinformatic analysis

#### Sequence alignment

Sequence reads were trimmed to remove transposon sequence and the remaining 12 bp sequences were aligned to the F11 genome consisting of 119 contigs (GenBank: AAJU00000000). The alignment was preformed with Bowtie [62], allowing no mismatches to the trimmed 12-mers. The number of times each 12-mer was observed (abundance) and the insertion position were tracked in a relational database. Because our strategy for determining candidate regions relied on the Wilcoxon signed-rank test, which is relatively resistant to spurious observations, there was no limit to how many times a given 12-mer was allowed to align to the genome.

#### Candidate identification

To distinguish loci within the F11 genome that were hypo or hyper-tolerant to transposon insertion, the average number of inserts observed within each annotated region, considering input pools 1, 2 and 3 alone, was calculated. A Z-test was performed on the population of 5,312 loci—those that fell below a standard score of −1 were considered hypo-tolerant and those that were above a standard score of +1 were considered hyper-tolerant. Standard scores were used here as an intuitive demarcation within the distribution of insertion tolerances and are not necessarily used to indicate any particular statistical significance. Of note, because we allowed reads to be aligned to the genome multiple times, we acknowledge that the specific values calculated for insertion tolerances for each annotated F11 region is overestimated. Indeed, we found that on average, reads could be aligned approximately twice to the F11 chromosome (distribution median = 2). This accounts for the elevated insertion rates reported in Figure 2 and Table S2, which are approximately two times greater than the inferred genome-wide insertion rate of 1 in 75 bp that is reported in the results section. Although this approach added a source of noise, it also allowed us to quantify and rank regions that would have otherwise been assigned a ‘0’ (i.e. putative essential genes). The design of future Tn-seq experiments should evaluate the pros and cons of such an approach.

To calculate changes in fitness *in vivo* due to transposon insertion, occurrence frequencies for insertion variants were first determined by normalizing insert abundance. The normalized occurrence rate of each insertion was obtained by dividing the number of times an insert was observed by the sum of insert counts for the insert positions that were detected in both the input and output pools of the replicate screen in question. Next, a Wilcoxon signed-rank test was performed on each F11 locus to compare the distribution of occurrence frequencies of inserts found within both input and respective output pools. Because this basic approach did not immediately account for inserts that were absent from output pools, which possibly indicates a strong negative selection event, we assigned the limit of detection (1/sequencing depth) to *lost* inserts that were present within a cognate input pool and within the top 1% of occurrence frequencies of all inserts that were *lost*. By incorporating these *lost* inserts prior to performing the Wilcoxon signed-rank test, we could more confidently account for inserts that likely represented a true negative selection signal while not considering inserts that were absent due to sampling error or passive loss. Regions having significantly altered frequency distributions (*p*<0.05) and at least 10 measured inserts were assigned to the *in piscis* gene set if the average occurrence frequency of inserts within the output samples was reduced by at least 2-fold, or assigned to the advantageous gene set if the average occurrence frequency of inserts within the output samples was greater than that of the input by at least 2-fold. Using these orthogonal vetting parameters we calculated that the false discovery rate (FDR), as defined by Benjamini and Hochberg, 1995, for the collection of observations with a maximal *p* value of 0.05 to be 0.096 and 0.094 for the *in piscis* and advantageous gene sets, respectively [63]. Figure S5 provides a diagrammatic summary of the bioinformatic pipeline used above to determine Tn-seq candidate genes and gene sets. Table S7, along with an online data viewer (http://pathogenomics.path.utah.edu/F11_TnSeq/), can be used to anonymously access and browse final metric-based data that were generated using these methods. Data analyses were done using custom software written in Python and the open source math tool SciPy.

#### KEGG-based functional analysis of gene sets

Kyoto Encyclopedia of Genes and Genomes (KEGG) orthologies (KO) were assigned to genes within the F11 genome using the KEGG automatic annotation server (KAAS) [64], [65], [66]. Genes without KO assignments were considered to have a ‘hypothetical’ function and genes encoding RNAs (e.g. tRNAs, non-coding RNAs and ribosomal RNAs) were manually assembled into a custom ‘RNA’ orthology. To test for enrichment of a particular KEGG functional category within a gene set, Monte Carlo simulations were performed by sampling an equivalent number of genes from the F11 genome at random 10,000 times without replacement. The functional composition of observed gene sets was then compared to theoretical sample sets using a Z-test. If a particular functional category was significantly overrepresented compared to chance (*p*<0.05) it was considered enriched. Data analyses were done using custom software written in Python and the open source math tool SciPy.

#### Trait enrichment analysis (TEA)

165 bacterial proteomes were downloaded from the National Center for Biotechnology Information's (NCBI) microbial genome sequence collection and assembled into the TEA metaproteome database (TEA-MD) (Table S3). Each strain included in the TEA-MD was annotated for habitat, niche of isolation, phylum and phenotype. Annotations were retrieved manually by referring to original sequencing project resources. Homologues within the TEA-MD were identified for each protein-coding gene within the F11 genome using the Basic Local Alignment Search Tool BLASTp [41]. Homologue assignment was conferred if bi-directional alignments produced an E value<10^−6^ with similarity over 50% of query length. The resulting set of TEA-MD microbes harboring homologues of specific F11 proteins were then used to determine particular trait associations. For each F11 protein, 1,000 Monte Carlo simulations were performed by sampling an equivalent number of bacterial genomes from the TEA-MD without replacement. Genes that were found to have significant trait enrichments (*p*<0.05) were also required to have at least 5 TEA-MD homologues, 60% of which belonged to pathogen genomes, to be further considered and placed into trait groups. TEA was performed in parallel and independent of Tn-seq. Cross-referencing of several well-known bacterial virulence factors, in addition to broadly conserved genes involved in basic bacterial physiology, confirmed that our TEA approach could reliably distinguish pathogenic identity between these two gene classes (Table S8). Data analyses were done using custom software written in Python and the open source math tool SciPy.

## Supporting Information

Figure S1 Plasmids used in this study. A) pSAM_Bt was originally constructed to express the *himar1C9* transposase (light green open reading frame) within *Bacteroides thetaiotaomicron*, facilitating insertion of the *ermG* erythromycin resistance gene (blue open reading frame) into the chromosome this bacterium. (B) pSAM_Bt was retrofitted using the indicated restriction sites with a kanamycin resistance gene (purple open reading frame) and a P*_lac_* promoter to drive expression of the *himar1C9* transposase within *E. coli*. This plasmid, pSAM-Ec, was then transferred into *E. coli* strain F11 via conjugation. Other pSAM-Ec features include: two transcriptional terminators downstream of the kanamycin resistance gene (red stop signs), P7 priming sites for Illumina sequencing (dark green blocks), MmeI-modified restriction sites (red blocks) flanking the transposon (grey), a β-lactamase gene for donor selection (red open reading frame) and an RP4 oriT/oriR6K origin of replication (orange block) for specific propagation in *pir*-positive donor strains.
(PDF)

Figure S2 Assessment of transposon mutant pool diversity and saturation. (A) *E. coli* F11 transposon mutants were randomly isolated and subjected to Southern blot analysis. Genomic DNA was isolated, digested with the restriction enzyme HindIII, resolved by gel electrophoresis and probed with a digoxigenin-labeled probe specific for the kanamycin resistance gene within the transposon. (B to D) Representative colorimetric microbiological agar plates were used to estimate mutant occurrence frequency within mutant pools. (B) *E. coli* F11 transposon mutants grown on MacConkey agar to assay for the presence of mutants deficient for lactose utilization (white colony within inset). (C) Agar plates containing the dye Congo red were used to determine the frequency of insertion variants that disrupted normal curli production. Inset shows a white colony (not able to bind Congo red due to the presumed absence of curli) surrounded by curli producing mutant variants (red colonies). (D) Mutant colonies grown on Kornberg agar and subsequently exposed to iodine vapor to stain for the presence of glycogen. Normal colonies exhibit a brown coloration, colonies lacking glycogen synthesis are yellow-white (left inset) and colonies overloaded with glycogen are dark brown or black (right inset).
(PDF)

Figure S3 Diagram of a 48 h post-fertilization zebrafish embryo. *E. coli* F11 transposon mutants were delivered into zebrafish embryos via one of two injections sites. The pericardial cavity (PC) simulates a localized infection—bacteria are restricted to the indicated area (green). The circulation valley (CV) is used to deliver bacteria systemically throughout the bloodstream (red).
(PDF)

Figure S4 Virulence of *E. coli* F11 within zebrafish embryos. (A) Approximately 2,000 colony-forming units of *E. coli* F11 were delivered into the pericardial cavity (PC, left) or the blood (right). Survival of embryos was monitored over a three-day period (*n* = 20 embryos for each curve). (B) At 21 h post-inoculation, infected zebrafish were homogenized and bacterial titers determined. Each square symbol represents an individual zebrafish and bars mark median values (*n = *10 embryos).
(PDF)

Figure S5 Description of pipeline used to define Tn-seq candidate gene sets. Diagram describes each of the five steps in determining Tn-seq-derived candidate genes and gene sets. (I) normalize insert read counts, (II) identify strong negative selection events and median-center output data, (III) perform Wilcoxon signed-rank test, (IV) initial candidate list vetting and (V) final gene set construction and curation.
(EPS)

Figure S6 Relative contributions made by each replicate screen to the *in piscis* gene set. For clarity, the *in piscis* gene set was divided into the component genes sets (A) blood, (B) PC and (C and D) multi-niche. The proportion of genes identified from only one replicate screen (numbered 1, 2 and 3) is represented by dark wedges, whereas colored wedges indicate genes that were identified from two or more screens using Tn-seq (bottom key). Fischer's exact test was used to determine if there was significant overlap between genes contributed by each replicate screen within each gene set (*p* values indicated where significance was found).
(PDF)

Figure S7 Compositional features of the TEA metaproteome database (TEA-MD). Pie graphs depict the proportion of protein sequences contained within the TEA-MD that are contributed by bacteria annotated with the indicated traits under the trait categories (A) habitat of isolation, (B) niche of isolation, (C) phylum and (D) phenotype. (E) Table detailing the total number of bacterial genomes and respective aggregate of protein sequences represented by specific traits within each trait category.
(PDF)

Figure S8 Comparison of phylum representation between NCBI and the TEA-MD. The number of genome sequences currently reported in the National Center for Biotechnology Information (NCBI) database for Proteobacteria, Bacteroidetes, Firmicutes, Actinobacteria, Chlamydiae and Cyanobacteria were aggregated and the resulting proportion for each phylum plotted (blue bars). In a similar manner, the proportion of genome sequences represented by each of the indicated phyla within the TEA-MD is plotted for comparison (gray bars).
(PDF)

Figure S9 *In vitro* growth kinetics of F11 mutants. (A) F11Δ*3256*, (B) F11Δ*3082*, (C) F11Δ*2628* and (D) F11Δ*3933* were grown in M9 minimal media shaking at the indicated temperatures. Optical density (y-axis) of the cultures was recorded over time (x-axis). Graphs are representative of at least three independent experiments performed in quadruplicate.
(PDF)

Protocol S1 Description of techniques carried out during this study.
(PDF)

Table S1 General sequence-based features of input and output pools.
(PDF)

Table S2 Insertion events per 100 bp for annotated regions within the F11 chromosome.
(XLSX)

Table S3 Listing of bacterial strains, accession numbers and traits maintained within the TEA-metaproteome database.
(XLSX)

Table S4 Composition of TEA-defined pathogen-associated genes within Tn-seq gene sets.
(PDF)

Table S5 Bacterial strains and plasmids.
(PDF)

Table S6 Primers used in this study.
(PDF)

Table S7 Raw calculated metrics used to rank fitness contributions for all annotated regions within F11 by Tn-seq.
(XLSX)

Table S8 TEA-assigned trait identities for known bacterial virulence factors and conserved genes involved in basic bacterial physiology.
(PDF)

## References

[1] Ewers C, Janssen T, Wieler LH (2003) [Avian pathogenic Escherichia coli (APEC)]. Berliner und Munchener tierarztliche Wochenschrift 116: 381–395. 14526468

[2] Shpigel NY, Elazar S, Rosenshine I (2008) Mammary pathogenic Escherichia coli. Current opinion in microbiology 11: 60–65. 18291708 10.1016/j.mib.2008.01.004

[3] Tan C, Xu Z, Zheng H, Liu W, Tang X, et al (2011) Genome sequence of a porcine extraintestinal pathogenic Escherichia coli strain. Journal of bacteriology 193: 5038. 21742868 10.1128/JB.05551-11 PMC3165708

[4] Carvallo FR, Debroy C, Baeza E, Hinckley L, Gilbert K, et al (2010) Necrotizing pneumonia and pleuritis associated with extraintestinal pathogenic Escherichia coli in a tiger (Panthera tigris) cub. Journal of veterinary diagnostic investigation : official publication of the American Association of Veterinary Laboratory Diagnosticians, Inc 22: 136–140. 10.1177/104063871002200130 20093704

[5] Foxman B (2010) The epidemiology of urinary tract infection. Nature reviews Urology 7: 653–660. 21139641 10.1038/nrurol.2010.190

[6] Johnson JR, Johnston B, Clabots C, Kuskowski MA, Castanheira M (2010) Escherichia coli sequence type ST131 as the major cause of serious multidrug-resistant E. coli infections in the United States. Clinical infectious diseases : an official publication of the Infectious Diseases Society of America 51: 286–294. 20572763 10.1086/653932

[7] Touchon M, Hoede C, Tenaillon O, Barbe V, Baeriswyl S, et al (2009) Organised genome dynamics in the Escherichia coli species results in highly diverse adaptive paths. PLoS genetics 5: e1000344. 19165319 10.1371/journal.pgen.1000344 PMC2617782

[8] Wiles TJ, Kulesus RR, Mulvey MA (2008) Origins and virulence mechanisms of uropathogenic Escherichia coli. Experimental and molecular pathology 85: 11–19. 18482721 10.1016/j.yexmp.2008.03.007 PMC2595135

[9] Muzzi A, Masignani V, Rappuoli R (2007) The pan-genome: towards a knowledge-based discovery of novel targets for vaccines and antibacterials. Drug discovery today 12: 429–439. 17532526 10.1016/j.drudis.2007.04.008

[10] Wiles TJ, Bower JM, Redd MJ, Mulvey MA (2009) Use of zebrafish to probe the divergent virulence potentials and toxin requirements of extraintestinal pathogenic Escherichia coli. PLoS pathogens 5: e1000697. 20019794 10.1371/journal.ppat.1000697 PMC2785880

[11] Wiles TJ, Norton JP, Smith SN, Lewis AJ, Mobley HL, et al (2013) A phyletically rare gene promotes the niche-specific fitness of an E. coli pathogen during bacteremia. PLoS pathogens 9: e1003175. 23459509 10.1371/journal.ppat.1003175 PMC3573123

[12] van Opijnen T, Camilli A (2012) A fine scale phenotype-genotype virulence map of a bacterial pathogen. Genome research 22: 2541–2551. 22826510 10.1101/gr.137430.112 PMC3514683

[13] Perez JC, Kumamoto CA, Johnson AD (2013) Candida albicans Commensalism and Pathogenicity Are Intertwined Traits Directed by a Tightly Knit Transcriptional Regulatory Circuit. PLoS biology 11: e1001510. 23526879 10.1371/journal.pbio.1001510 PMC3601966

[14] van Opijnen T, Bodi KL, Camilli A (2009) Tn-seq: high-throughput parallel sequencing for fitness and genetic interaction studies in microorganisms. Nature methods 6: 767–772. 19767758 10.1038/nmeth.1377 PMC2957483

[15] Goodman AL, McNulty NP, Zhao Y, Leip D, Mitra RD, et al (2009) Identifying genetic determinants needed to establish a human gut symbiont in its habitat. Cell host & microbe 6: 279–289. 19748469 10.1016/j.chom.2009.08.003 PMC2895552

[16] Joshi SM, Pandey AK, Capite N, Fortune SM, Rubin EJ, et al (2006) Characterization of mycobacterial virulence genes through genetic interaction mapping. Proceedings of the National Academy of Sciences of the United States of America 103: 11760–11765. 16868085 10.1073/pnas.0603179103 PMC1544243

[17] Hensel M, Shea JE, Gleeson C, Jones MD, Dalton E, et al (1995) Simultaneous identification of bacterial virulence genes by negative selection. Science 269: 400–403. 7618105 10.1126/science.7618105

[18] Saenz HL, Dehio C (2005) Signature-tagged mutagenesis: technical advances in a negative selection method for virulence gene identification. Current opinion in microbiology 8: 612–619. 16126452 10.1016/j.mib.2005.08.013

[19] Hamer L, DeZwaan TM, Montenegro-Chamorro MV, Frank SA, Hamer JE (2001) Recent advances in large-scale transposon mutagenesis. Current opinion in chemical biology 5: 67–73. 11166651 10.1016/s1367-5931(00)00162-9

[20] Lehoux DE, Sanschagrin F, Levesque RC (2001) Discovering essential and infection-related genes. Current opinion in microbiology 4: 515–519. 11587926 10.1016/s1369-5274(00)00244-7

[21] Hayes F (2003) Transposon-based strategies for microbial functional genomics and proteomics. Annual review of genetics 37: 3–29. 10.1146/annurev.genet.37.110801.142807 14616054

[22] Trede NS, Langenau DM, Traver D, Look AT, Zon LI (2004) The use of zebrafish to understand immunity. Immunity 20: 367–379. 15084267 10.1016/s1074-7613(04)00084-6

[23] Jault C, Pichon L, Chluba J (2004) Toll-like receptor gene family and TIR-domain adapters in Danio rerio. Molecular immunology 40: 759–771. 14687933 10.1016/j.molimm.2003.10.001

[24] Wang Z, Zhang S, Wang G, An Y (2008) Complement activity in the egg cytosol of zebrafish Danio rerio: evidence for the defense role of maternal complement components. PloS one 3: e1463. 18213372 10.1371/journal.pone.0001463 PMC2194919

[25] Li X, Wang S, Qi J, Echtenkamp SF, Chatterjee R, et al (2007) Zebrafish peptidoglycan recognition proteins are bactericidal amidases essential for defense against bacterial infections. Immunity 27: 518–529. 17892854 10.1016/j.immuni.2007.07.020 PMC2074879

[26] Lieschke GJ, Oates AC, Crowhurst MO, Ward AC, Layton JE (2001) Morphologic and functional characterization of granulocytes and macrophages in embryonic and adult zebrafish. Blood 98: 3087–3096. 11698295 10.1182/blood.v98.10.3087

[27] Lampe DJ, Churchill ME, Robertson HM (1996) A purified mariner transposase is sufficient to mediate transposition in vitro. The EMBO journal 15: 5470–5479. 8895590 PMC452289

[28] Smith V, Botstein D, Brown PO (1995) Genetic footprinting: a genomic strategy for determining a gene's function given its sequence. Proceedings of the National Academy of Sciences of the United States of America 92: 6479–6483. 7604017 10.1073/pnas.92.14.6479 PMC41541

[29] Smith V, Chou KN, Lashkari D, Botstein D, Brown PO (1996) Functional analysis of the genes of yeast chromosome V by genetic footprinting. Science 274: 2069–2074. 8953036 10.1126/science.274.5295.2069

[30] Grant AJ, Farris M, Alefounder P, Williams PH, Woodward MJ, et al (2003) Co-ordination of pathogenicity island expression by the BipA GTPase in enteropathogenic Escherichia coli (EPEC). Molecular microbiology 48: 507–521. 12675808 10.1046/j.1365-2958.2003.t01-1-03447.x

[31] Iyoda S, Honda N, Saitoh T, Shimuta K, Terajima J, et al (2011) Coordinate control of the locus of enterocyte effacement and enterohemolysin genes by multiple common virulence regulators in enterohemorrhagic Escherichia coli. Infection and immunity 79: 4628–4637. 21844237 10.1128/IAI.05023-11 PMC3257950

[32] Nagy G, Dobrindt U, Schneider G, Khan AS, Hacker J, et al (2002) Loss of regulatory protein RfaH attenuates virulence of uropathogenic Escherichia coli. Infection and immunity 70: 4406–4413. 12117951 10.1128/IAI.70.8.4406-4413.2002 PMC128157

[33] Burns SM, Hull SI (1999) Loss of resistance to ingestion and phagocytic killing by O(−) and K(−) mutants of a uropathogenic Escherichia coli O75:K5 strain. Infection and immunity 67: 3757–3762. 10417134 10.1128/iai.67.8.3757-3762.1999 PMC96650

[34] Buckles EL, Wang X, Lane MC, Lockatell CV, Johnson DE, et al (2009) Role of the K2 capsule in Escherichia coli urinary tract infection and serum resistance. The Journal of infectious diseases 199: 1689–1697. 19432551 10.1086/598524 PMC3872369

[35] deLivron MA, Robinson VL (2008) Salmonella enterica serovar Typhimurium BipA exhibits two distinct ribosome binding modes. Journal of bacteriology 190: 5944–5952. 18621905 10.1128/JB.00763-08 PMC2519513

[36] Kiss E, Huguet T, Poinsot V, Batut J (2004) The typA gene is required for stress adaptation as well as for symbiosis of Sinorhizobium meliloti 1021 with certain Medicago truncatula lines. Molecular plant-microbe interactions : MPMI 17: 235–244. 15000390 10.1094/MPMI.2004.17.3.235

[37] Gauger EJ, Leatham MP, Mercado-Lubo R, Laux DC, Conway T, et al (2007) Role of motility and the flhDC Operon in Escherichia coli MG1655 colonization of the mouse intestine. Infection and immunity 75: 3315–3324. 17438023 10.1128/IAI.00052-07 PMC1932950

[38] Morris JJ, Lenski RE, Zinser ER (2012) The Black Queen Hypothesis: evolution of dependencies through adaptive gene loss. mBio 3: e00036–12. 22448042 10.1128/mBio.00036-12 PMC3315703

[39] Falkow S (2004) Molecular Koch's postulates applied to bacterial pathogenicity–a personal recollection 15 years later. Nature reviews Microbiology 2: 67–72. 15035010 10.1038/nrmicro799

[40] Falkow S (1988) Molecular Koch's postulates applied to microbial pathogenicity. Reviews of infectious diseases 10 Suppl 2: S274–276. 3055197 10.1093/cid/10.supplement_2.s274

[41] Altschul SF, Madden TL, Schaffer AA, Zhang J, Zhang Z, et al (1997) Gapped BLAST and PSI-BLAST: a new generation of protein database search programs. Nucleic acids research 25: 3389–3402. 9254694 10.1093/nar/25.17.3389 PMC146917

[42] del Castillo I, Gonzalez-Pastor JE, San Millan JL, Moreno F (1991) Nucleotide sequence of the Escherichia coli regulatory gene mprA and construction and characterization of mprA-deficient mutants. Journal of bacteriology 173: 3924–3929. 1840583 10.1128/jb.173.12.3924-3929.1991 PMC208030

[43] Lomovskaya O, Lewis K, Matin A (1995) EmrR is a negative regulator of the Escherichia coli multidrug resistance pump EmrAB. Journal of bacteriology 177: 2328–2334. 7730261 10.1128/jb.177.9.2328-2334.1995 PMC176888

[44] Kelley LA, Sternberg MJ (2009) Protein structure prediction on the Web: a case study using the Phyre server. Nature protocols 4: 363–371. 19247286 10.1038/nprot.2009.2

[45] Arnadottir H, Hvanndal I andresdottir V, Burr SE, Frey J, et al (2009) The AsaP1 peptidase of Aeromonas salmonicida subsp. achromogenes is a highly conserved deuterolysin metalloprotease (family M35) and a major virulence factor. Journal of bacteriology 191: 403–410. 18952802 10.1128/JB.00847-08 PMC2612443

[46] Tadesse S, Graumann PL (2007) DprA/Smf protein localizes at the DNA uptake machinery in competent Bacillus subtilis cells. BMC microbiology 7: 105. 18045469 10.1186/1471-2180-7-105 PMC2216020

[47] Karudapuram S, Zhao X, Barcak GJ (1995) DNA sequence and characterization of Haemophilus influenzae dprA+, a gene required for chromosomal but not plasmid DNA transformation. Journal of bacteriology 177: 3235–3240. 7768823 10.1128/jb.177.11.3235-3240.1995 PMC177016

[48] Smeets LC, Becker SC, Barcak GJ, Vandenbroucke-Grauls CM, Bitter W, et al (2006) Functional characterization of the competence protein DprA/Smf in Escherichia coli. FEMS Microbiology Letters 263: 223–228. 16978360 10.1111/j.1574-6968.2006.00423.x

[49] Norton JP, Mulvey MA (2012) Toxin-Antitoxin Systems Are Important for Niche-Specific Colonization and Stress Resistance of Uropathogenic Escherichia coli. PLoS pathogens 8: e1002954. 23055930 10.1371/journal.ppat.1002954 PMC3464220

[50] Smith SN, Hagan EC, Lane MC, Mobley HL (2010) Dissemination and systemic colonization of uropathogenic Escherichia coli in a murine model of bacteremia. mBio 1: e00262–10. 21116344 10.1128/mBio.00262-10 PMC2993011

[51] Welch RA, Dellinger EP, Minshew B, Falkow S (1981) Haemolysin contributes to virulence of extra-intestinal E. coli infections. Nature 294: 665–667. 7031483 10.1038/294665a0

[52] Picard B, Garcia JS, Gouriou S, Duriez P, Brahimi N, et al (1999) The link between phylogeny and virulence in Escherichia coli extraintestinal infection. Infection and immunity 67: 546–553. 9916057 10.1128/iai.67.2.546-553.1999 PMC96353

[53] Pfennig PL, Flower AM (2001) BipA is required for growth of Escherichia coi K12 at low temperature. Molecular genetics and genomics : MGG 266: 313–317. 11683274 10.1007/s004380100559

[54] Krin E, Danchin A, Soutourina O (2010) Decrypting the H-NS-dependent regulatory cascade of acid stress resistance in Escherichia coli. BMC microbiology 10: 273. 21034467 10.1186/1471-2180-10-273 PMC2984483

[55] Richards GR, Herbert EE, Park Y, Goodrich-Blair H (2008) Xenorhabdus nematophila lrhA is necessary for motility, lipase activity, toxin expression and virulence in Manduca sexta insects. Journal of bacteriology 190: 4870–4879. 18502863 10.1128/JB.00358-08 PMC2447004

[56] Himpsl SD, Lockatell CV, Hebel JR, Johnson DE, Mobley HL (2008) Identification of virulence determinants in uropathogenic Proteus mirabilis using signature-tagged mutagenesis. Journal of medical microbiology 57: 1068–1078. 18719175 10.1099/jmm.0.2008/002071-0

[57] Whitfield C (2006) Biosynthesis and assembly of capsular polysaccharides in Escherichia coli. Annual review of biochemistry 75: 39–68. 10.1146/annurev.biochem.75.103004.142545 16756484

[58] Price PA, Jin J, Goldman WE (2012) Pulmonary infection by Yersinia pestis rapidly establishes a permissive environment for microbial proliferation. Proceedings of the National Academy of Sciences of the United States of America 109: 3083–3088. 22308352 10.1073/pnas.1112729109 PMC3286930

[59] Hagan EC, Lloyd AL, Rasko DA, Faerber GJ, Mobley HL (2010) Escherichia coli global gene expression in urine from women with urinary tract infection. PLoS pathogens 6: e1001187. 21085611 10.1371/journal.ppat.1001187 PMC2978726

[60] Datsenko KA, Wanner BL (2000) One-step inactivation of chromosomal genes in Escherichia coli K-12 using PCR products. Proceedings of the National Academy of Sciences of the United States of America 97: 6640–6645. 10829079 10.1073/pnas.120163297 PMC18686

[61] Murphy KC, Campellone KG (2003) Lambda Red-mediated recombinogenic engineering of enterohemorrhagic and enteropathogenic E. coli. BMC molecular biology 4: 11. 14672541 10.1186/1471-2199-4-11 PMC317293

[62] Langmead B, Trapnell C, Pop M, Salzberg SL (2009) Ultrafast and memory-efficient alignment of short DNA sequences to the human genome. Genome biology 10: R25. 19261174 10.1186/gb-2009-10-3-r25 PMC2690996

[63] Benjamini Y, Hochberg Y (1995) Controlling the False Discovery Rate: a Practical and Powerful Approach to Multiple Testing. Journal of the Royal Statistical Society, Series B 57: 289–300.

[64] Kanehisa M, Goto S (2000) KEGG: kyoto encyclopedia of genes and genomes. Nucleic acids research 28: 27–30. 10592173 10.1093/nar/28.1.27 PMC102409

[65] Kanehisa M, Goto S, Sato Y, Furumichi M, Tanabe M (2012) KEGG for integration and interpretation of large-scale molecular data sets. Nucleic acids research 40: D109–114. 22080510 10.1093/nar/gkr988 PMC3245020

[66] Moriya Y, Itoh M, Okuda S, Yoshizawa AC, Kanehisa M (2007) KAAS: an automatic genome annotation and pathway reconstruction server. Nucleic acids research 35: W182–185. 17526522 10.1093/nar/gkm321 PMC1933193

[67] Rakeman JL, Bonifield HR, Miller SI (1999) A HilA-independent pathway to Salmonella typhimurium invasion gene transcription. Journal of bacteriology 181: 3096–3104. 10322010 10.1128/jb.181.10.3096-3104.1999 PMC93764

[68] Jakomin M, Chessa D, Baumler AJ, Casadesus J (2008) Regulation of the Salmonella enterica std fimbrial operon by DNA adenine methylation, SeqA and HdfR. Journal of bacteriology 190: 7406–7413. 18805972 10.1128/JB.01136-08 PMC2576648

[69] Ko M, Park C (2000) H-NS-Dependent regulation of flagellar synthesis is mediated by a LysR family protein. Journal of bacteriology 182: 4670–4672. 10913108 10.1128/jb.182.16.4670-4672.2000 PMC94646

[70] Pavelka MS Jr, Wright LF, Silver RP (1991) Identification of two genes, kpsM and kpsT, in region 3 of the polysialic acid gene cluster of Escherichia coli K1. Journal of bacteriology 173: 4603–4610. 1856162 10.1128/jb.173.15.4603-4610.1991 PMC208135

[71] Jakobsen L, Spangholm DJ, Pedersen K, Jensen LB, Emborg HD, et al (2010) Broiler chickens, broiler chicken meat, pigs and pork as sources of ExPEC related virulence genes and resistance in Escherichia coli isolates from community-dwelling humans and UTI patients. International journal of food microbiology 142: 264–272. 20656368 10.1016/j.ijfoodmicro.2010.06.025

[72] Rigg GP, Barrett B, Roberts IS (1998) The localization of KpsC, S and T and KfiA, C and D proteins involved in the biosynthesis of the Escherichia coli K5 capsular polysaccharide: evidence for a membrane-bound complex. Microbiology 144 Pt 10: 2905–2914. 9802032 10.1099/00221287-144-10-2905

[73] Bronner D, Sieberth V, Pazzani C, Roberts IS, Boulnois GJ, et al (1993) Expression of the capsular K5 polysaccharide of Escherichia coli: biochemical and electron microscopic analyses of mutants with defects in region 1 of the K5 gene cluster. Journal of bacteriology 175: 5984–5992. 8397188 10.1128/jb.175.18.5984-5992.1993 PMC206680

[74] McNulty C, Thompson J, Barrett B, Lord L andersen C, et al (2006) The cell surface expression of group 2 capsular polysaccharides in Escherichia coli: the role of KpsD, RhsA and a multi-protein complex at the pole of the cell. Molecular microbiology 59: 907–922. 16420360 10.1111/j.1365-2958.2005.05010.x

[75] Bachtiar BM, Coloe PJ, Fry BN (2007) Knockout mutagenesis of the kpsE gene of Campylobacter jejuni 81116 and its involvement in bacterium-host interactions. FEMS immunology and medical microbiology 49: 149–154. 17266722 10.1111/j.1574-695X.2006.00182.x

[76] Cieslewicz M, Vimr E (1997) Reduced polysialic acid capsule expression in Escherichia coli K1 mutants with chromosomal defects in kpsF. Molecular microbiology 26: 237–249. 9383150 10.1046/j.1365-2958.1997.5651942.x

[77] Kalynych S, Ruan X, Valvano MA, Cygler M (2011) Structure-guided investigation of lipopolysaccharide O-antigen chain length regulators reveals regions critical for modal length control. Journal of bacteriology 193: 3710–3721. 21642455 10.1128/JB.00059-11 PMC3147518

[78] Amos MR, Sanchez-Contreras M, Jackson RW, Munoz-Berbel X, Ciche TA, et al (2011) Influence of the Photorhabdus luminescens phosphomannose isomerase gene, manA, on mannose utilization, exopolysaccharide structure and biofilm formation. Applied and environmental microbiology 77: 776–785. 21148694 10.1128/AEM.02326-10 PMC3028718

[79] Ouyang Z, Isaacson R (2006) Identification and characterization of a novel ABC iron transport system, fit, in Escherichia coli. Infection and immunity 74: 6949–6956. 16982838 10.1128/IAI.00866-06 PMC1698097

[80] Stojiljkovic I, Baumler AJ, Heffron F (1995) Ethanolamine utilization in Salmonella typhimurium: nucleotide sequence, protein expression and mutational analysis of the cchA cchB eutE eutJ eutG eutH gene cluster. Journal of bacteriology 177: 1357–1366. 7868611 10.1128/jb.177.5.1357-1366.1995 PMC176743

[81] Buan NR, Suh SJ, Escalante-Semerena JC (2004) The eutT gene of Salmonella enterica Encodes an oxygen-labile, metal-containing ATP:corrinoid adenosyltransferase enzyme. Journal of bacteriology 186: 5708–5714. 15317775 10.1128/JB.186.17.5708-5714.2004 PMC516830

[82] Turner RJ, Taylor DE, Weiner JH (1997) Expression of Escherichia coli TehA gives resistance to antiseptics and disinfectants similar to that conferred by multidrug resistance efflux pumps. Antimicrobial agents and chemotherapy 41: 440–444. 9021204 10.1128/aac.41.2.440 PMC163726

[83] Nataro JP, Seriwatana J, Fasano A, Maneval DR, Guers LD, et al (1995) Identification and cloning of a novel plasmid-encoded enterotoxin of enteroinvasive Escherichia coli and Shigella strains. Infection and immunity 63: 4721–4728. 7591128 10.1128/iai.63.12.4721-4728.1995 PMC173677

[84] Schwan WR (2009) Survival of uropathogenic Escherichia coli in the murine urinary tract is dependent on OmpR. Microbiology 155: 1832–1839. 19383700 10.1099/mic.0.026187-0 PMC2888292

[85] Ware D, Jiang Y, Lin W, Swiatlo E (2006) Involvement of potD in Streptococcus pneumoniae polyamine transport and pathogenesis. Infection and immunity 74: 352–361. 16368990 10.1128/IAI.74.1.352-361.2006 PMC1346612

