# Correction: Combining Quantitative Genetic Footprinting and Trait Enrichment Analysis to Identify Fitness Determinants of a Bacterial Pathogen

**DOI:** 10.1371/annotation/6bb09d48-7d06-4ccf-8ed1-3dfaa1e0d537

**Published:** 2013-09-06

**Authors:** Travis J. Wiles, J. Paul Norton, Colin W. Russell, Brian K. Dalley, Kael F. Fischer, Matthew A. Mulvey

Figure S5 is a duplicate of Figure 5. Please view the correct Figure S5 here: 
