# Supplementary figures and images for: Combining Quantitative Genetic Footprinting and Trait Enrichment Analysis to Identify Fitness Determinants of a Bacterial Pathogen
Source: PLoS Genet. 2013 Aug 22;9(8):e1003716. doi: 10.1371/journal.pgen.1003716 (PMC3749937; doi:10.1371/journal.pgen.1003716)

**A**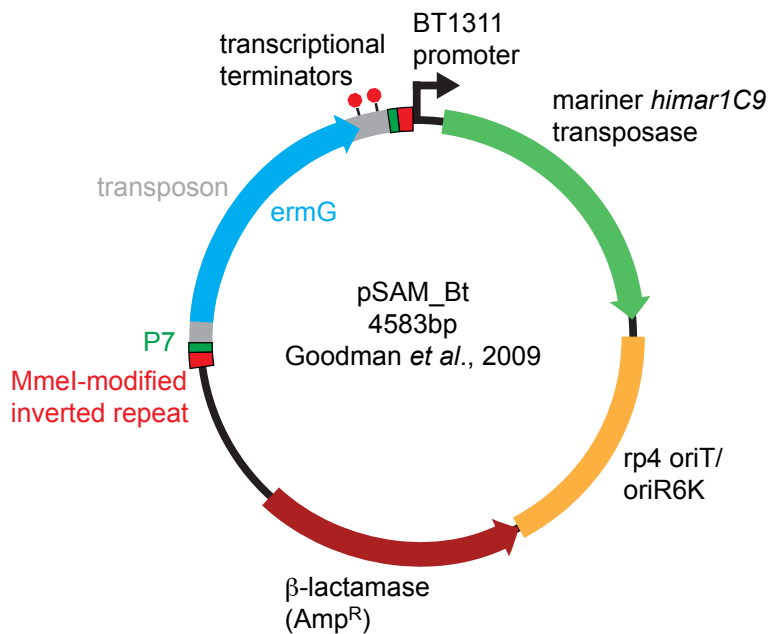**B**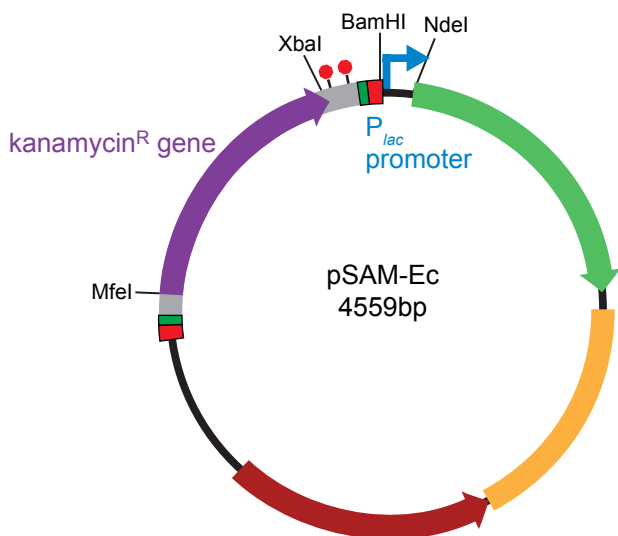

Supplement: Figure S1 — Plasmids used in this study. A) pSAM_Bt was originally constructed to express the himar1C9 transposase (light green open reading frame) within Bacteroides thetaiotaomicron, facilitating insertion of the ermG erythromycin resistance gene (blue open reading frame) into the chromosome this bacterium. (B) pSAM_Bt was retrofitted using the indicated restriction sites with a kanamycin resistance gene (purple open reading frame) and a Plac promoter to drive expression of the himar1C9 transposase within E. coli. This plasmid, pSAM-Ec, was then transferred into E. coli strain F11 via conjugation. Other pSAM-Ec features include: two transcriptional terminators downstream of the kanamycin resistance gene (red stop signs), P7 priming sites for Illumina sequencing (dark green blocks), MmeI-modified restriction sites (red blocks) flanking the transposon (grey), a β-lactamase gene for donor selection (red open reading frame) and an RP4 oriT/oriR6K origin of replication (orange block) for specific propagation in pir-positive donor strains. (PDF) [file pgen.1003716.s001.pdf]

**A**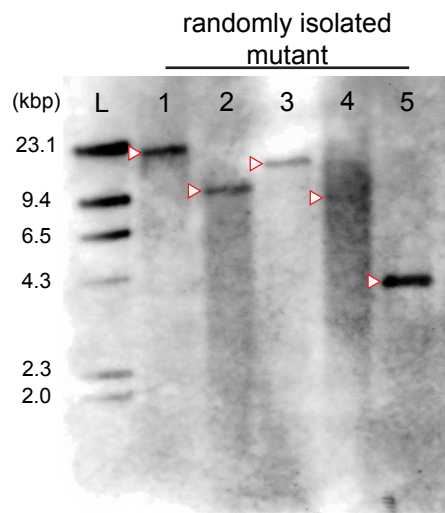**B**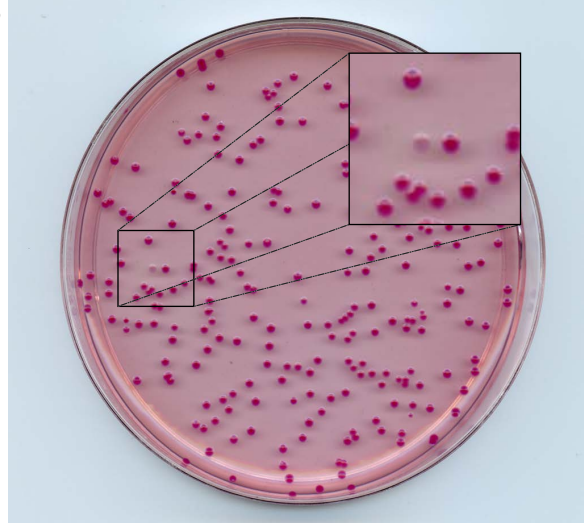**C**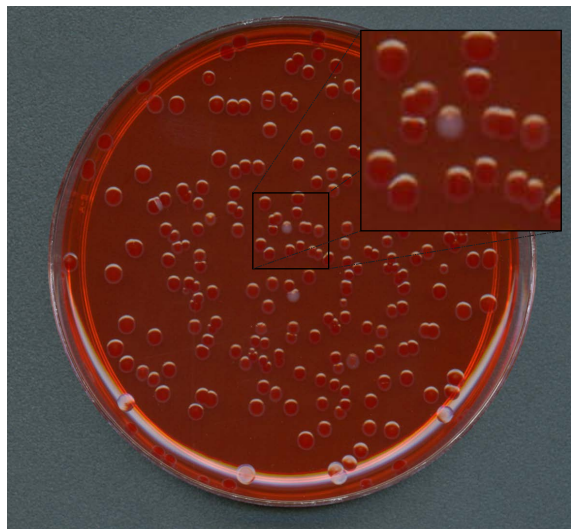**D**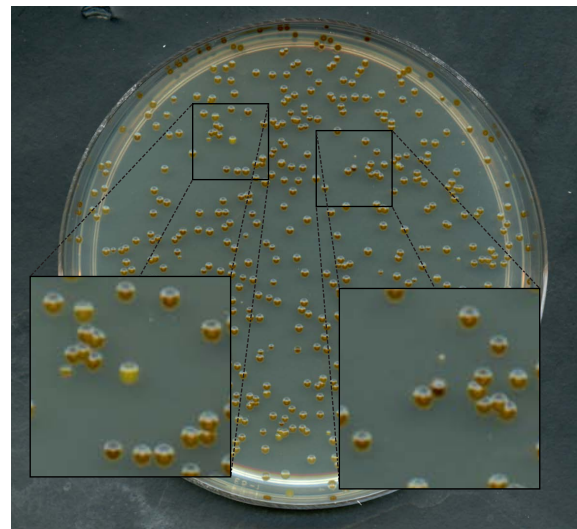

Supplement: Figure S2 — Assessment of transposon mutant pool diversity and saturation. (A) E. coli F11 transposon mutants were randomly isolated and subjected to Southern blot analysis. Genomic DNA was isolated, digested with the restriction enzyme HindIII, resolved by gel electrophoresis and probed with a digoxigenin-labeled probe specific for the kanamycin resistance gene within the transposon. (B to D) Representative colorimetric microbiological agar plates were used to estimate mutant occurrence frequency within mutant pools. (B) E. coli F11 transposon mutants grown on MacConkey agar to assay for the presence of mutants deficient for lactose utilization (white colony within inset). (C) Agar plates containing the dye Congo red were used to determine the frequency of insertion variants that disrupted normal curli production. Inset shows a white colony (not able to bind Congo red due to the presumed absence of curli) surrounded by curli producing mutant variants (red colonies). (D) Mutant colonies grown on Kornberg agar and subsequently exposed to iodine vapor to stain for the presence of glycogen. Normal colonies exhibit a brown coloration, colonies lacking glycogen synthesis are yellow-white (left inset) and colonies overloaded with glycogen are dark brown or black (right inset). (PDF) [file pgen.1003716.s002.pdf]

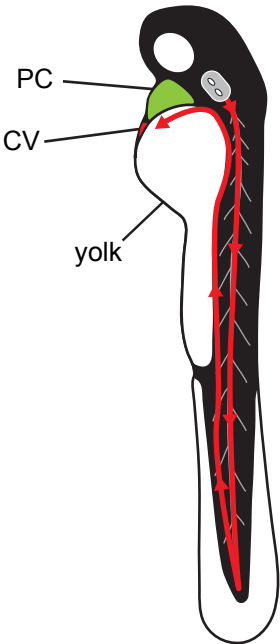

Supplement: Figure S3 — Diagram of a 48 h post-fertilization zebrafish embryo. E. coli F11 transposon mutants were delivered into zebrafish embryos via one of two injections sites. The pericardial cavity (PC) simulates a localized infection—bacteria are restricted to the indicated area (green). The circulation valley (CV) is used to deliver bacteria systemically throughout the bloodstream (red). (PDF) [file pgen.1003716.s003.pdf]

**A**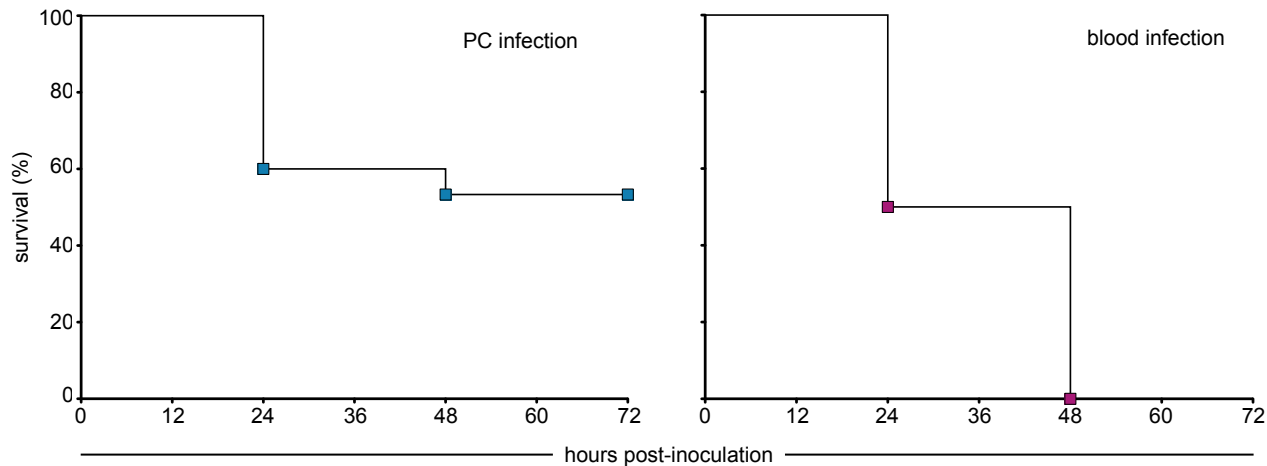**B**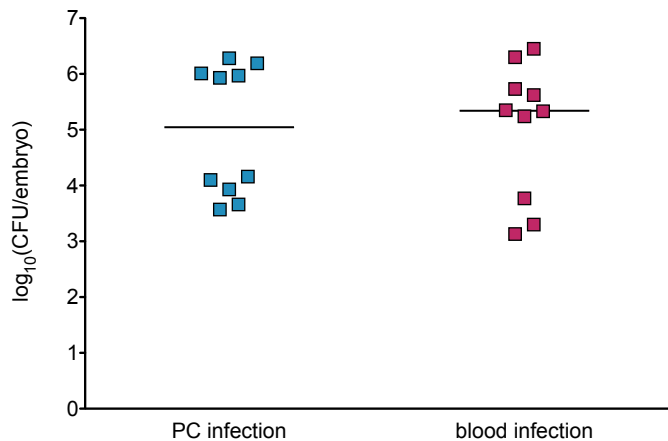

Supplement: Figure S4 — Virulence of E. coli F11 within zebrafish embryos. (A) Approximately 2,000 colony-forming units of E. coli F11 were delivered into the pericardial cavity (PC, left) or the blood (right). Survival of embryos was monitored over a three-day period (n = 20 embryos for each curve). (B) At 21 h post-inoculation, infected zebrafish were homogenized and bacterial titers determined. Each square symbol represents an individual zebrafish and bars mark median values (n = 10 embryos). (PDF) [file pgen.1003716.s004.pdf]

**A**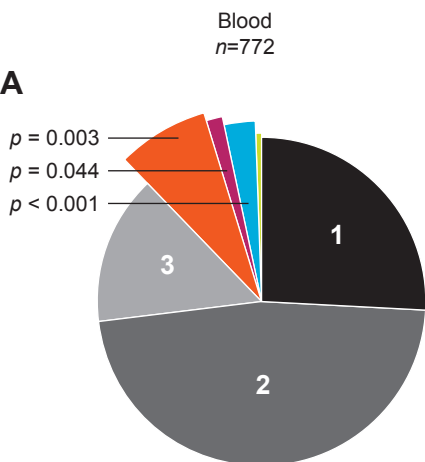**B**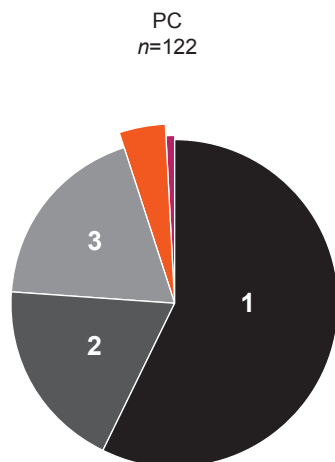**C**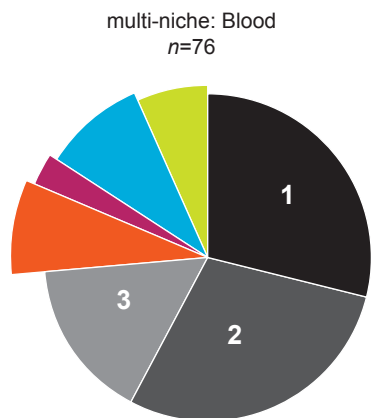**D**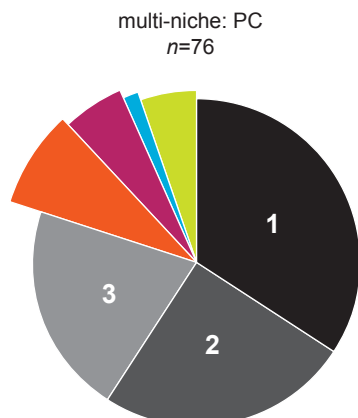

replicate 1

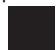

replicate 2

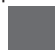

replicate 3

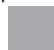

1+2

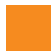

1+3

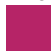

2+3

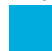

1+2+3

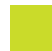

Supplement: Figure S6 — Relative contributions made by each replicate screen to the in piscis gene set. For clarity, the in piscis gene set was divided into the component genes sets (A) blood, (B) PC and (C and D) multi-niche. The proportion of genes identified from only one replicate screen (numbered 1, 2 and 3) is represented by dark wedges, whereas colored wedges indicate genes that were identified from two or more screens using Tn-seq (bottom key). Fischer's exact test was used to determine if there was significant overlap between genes contributed by each replicate screen within each gene set (p values indicated where significance was found). (PDF) [file pgen.1003716.s006.pdf]

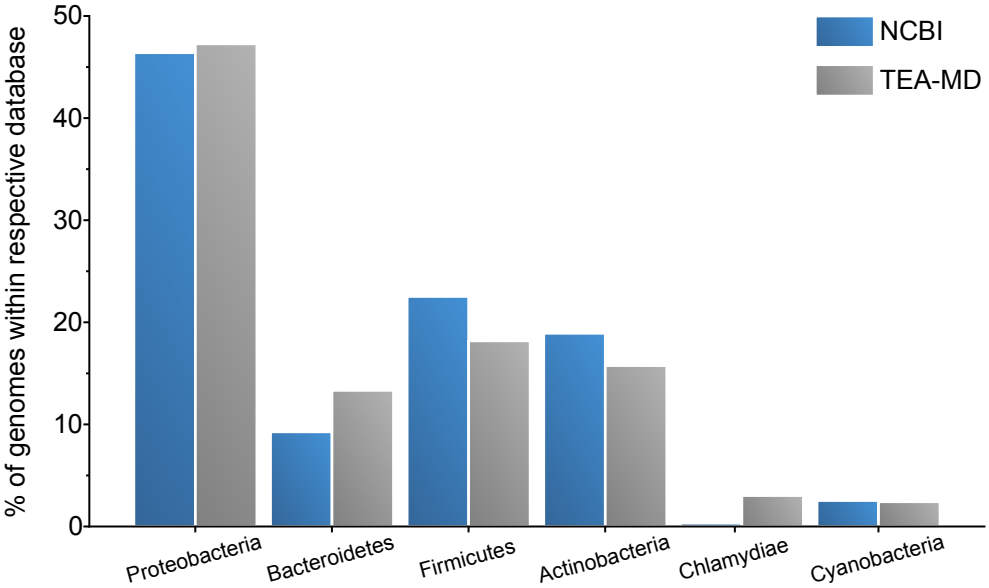

Supplement: Figure S8 — Comparison of phylum representation between NCBI and the TEA-MD. The number of genome sequences currently reported in the National Center for Biotechnology Information (NCBI) database for Proteobacteria, Bacteroidetes, Firmicutes, Actinobacteria, Chlamydiae and Cyanobacteria were aggregated and the resulting proportion for each phylum plotted (blue bars). In a similar manner, the proportion of genome sequences represented by each of the indicated phyla within the TEA-MD is plotted for comparison (gray bars). (PDF) [file pgen.1003716.s008.pdf]

**A**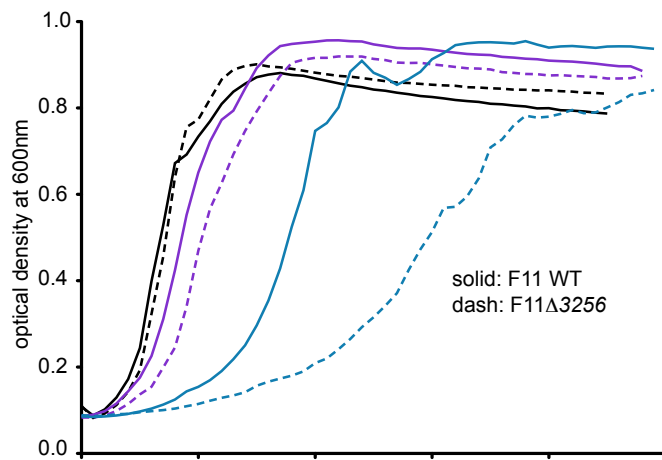**B**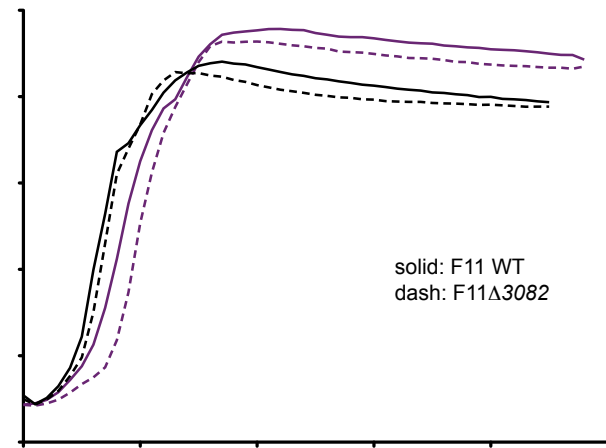**C**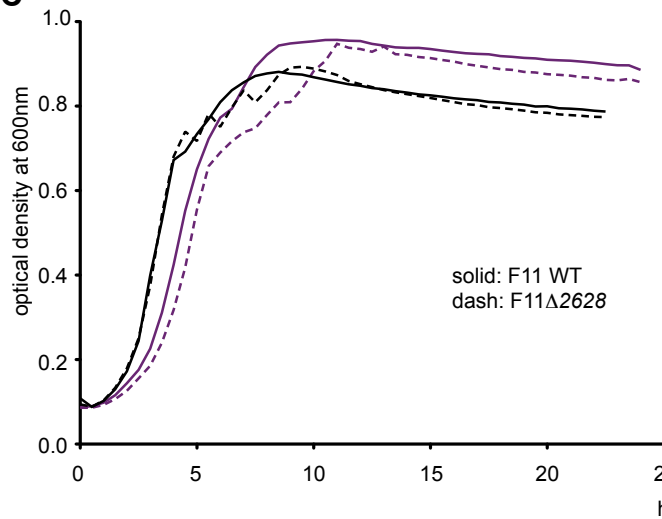**D**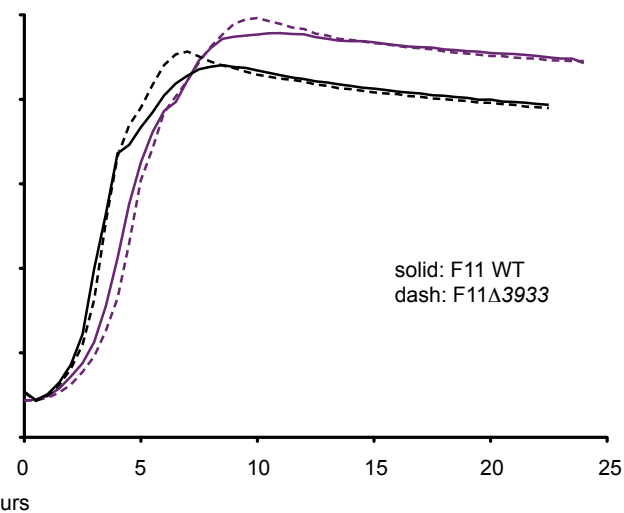

37°C 28.5°C 20°C

Supplement: Figure S9 — In vitro growth kinetics of F11 mutants. (A) F11Δ3256, (B) F11Δ3082, (C) F11Δ2628 and (D) F11Δ3933 were grown in M9 minimal media shaking at the indicated temperatures. Optical density (y-axis) of the cultures was recorded over time (x-axis). Graphs are representative of at least three independent experiments performed in quadruplicate. (PDF) [file pgen.1003716.s009.pdf]
