# Supplementary material for: Combining Quantitative Genetic Footprinting and Trait Enrichment Analysis to Identify Fitness Determinants of a Bacterial Pathogen
Source: PLoS Genet. 2013 Aug 22;9(8):e1003716. doi: 10.1371/journal.pgen.1003716 (PMC3749937; doi:10.1371/journal.pgen.1003716)
Supplement: Figure S7 — Compositional features of the TEA metaproteome database (TEA-MD). Pie graphs depict the proportion of protein sequences contained within the TEA-MD that are contributed by bacteria annotated with the indicated traits under the trait categories (A) habitat of isolation, (B) niche of isolation, (C) phylum and (D) phenotype. (E) Table detailing the total number of bacterial genomes and respective aggregate of protein sequences represented by specific traits within each trait category. (PDF) [file pgen.1003716.s007.pdf]

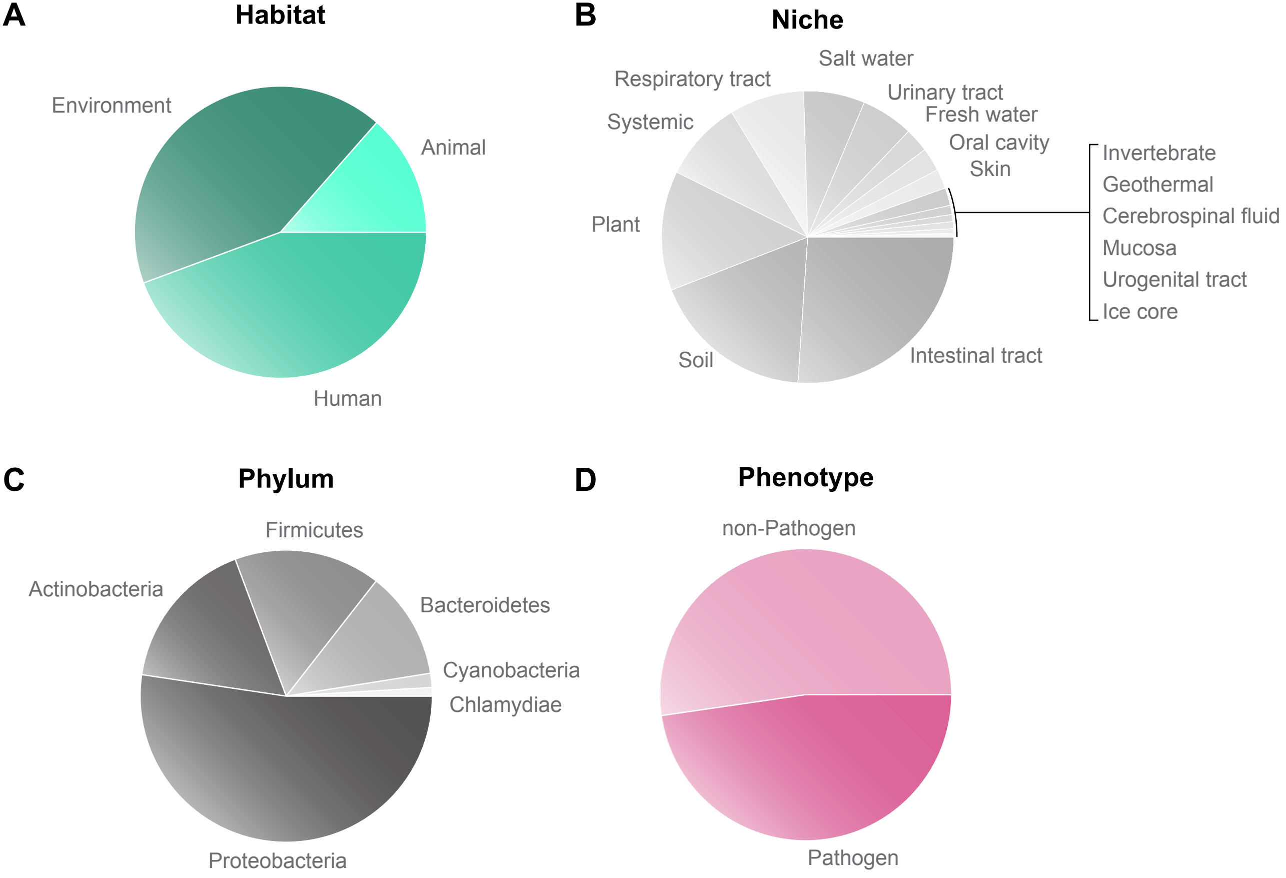

E

**Habitat**	total genomes	total proteins
Animal	26	83,103
Environment	59	258,787
Human	80	272,437
**Niche**		
Ice core	1	2,534
Urogenital tract	2	3,172
Mucosa	4	4,753
Cerebrospinal fluid	1	4,827
Geothermal	2	6,178
Invertebrate	5	12,071
Skin	4	13,236
Oral cavity	8	15,903
Fresh water	4	16,455
Urinary tract	8	35,554
Salt Water	12	41,606
Respiratory tract	14	50,777
Systemic	19	55,106
Plant	17	81,255

**Niche** *continued*	total genomes	total proteins
Soil	23	110,759
Intestinal tract	41	160,141
**Phylum**		
Chlamydiae	5	5,822
Cyanobacteria	4	9,596
Bacteroidetes	22	73,311
Firmicutes	30	99,767
Actinobacteria	26	104,210
Proteobacteria	78	321,621
**Phenotype**		
non-Pathogen	81	321,251
Pathogen	84	293,076
