# Supplementary material for: Combining Quantitative Genetic Footprinting and Trait Enrichment Analysis to Identify Fitness Determinants of a Bacterial Pathogen
Source: PLoS Genet. 2013 Aug 22;9(8):e1003716. doi: 10.1371/journal.pgen.1003716 (PMC3749937; doi:10.1371/journal.pgen.1003716)
Supplement: Protocol S1 — Description of techniques carried out during this study. (PDF) [file pgen.1003716.s010.pdf]

Combining Quantitative Genetic Footprinting and Trait Enrichment Analysis to Identify Fitness Determinants of a Bacterial Pathogen

Wiles *et al.*

Protocol S1 contents:

- *Transposon mutagenesis*
- *Southern blotting*
- *Plating assays*
- *Preparation of excised transposons for indexed sequencing*

Transposon Mutagenesis

Transposon mutagenesis using EcS17 donor strain: Auxotrophies- Thi, Pro

Media required:

1. Wash media: 1x M9 salts (no supplements except $MgSO_4$ and $CaCl_2$)
2. Pre-mating media: 1x M9-thr-pro-thi-glucose broth
3. Mating agar: 1x M9-thr-pro-thi-glucose agar
4. Selective agar: 1x M9-thr-glucose-kan agar
5. Selective media: 1x M9-thr-glucose broth (add kanamycin to select for transposon mutants when needed)

[notes on media prep]

- M9 salts: 6 g/L Na_2HPO_4, 3 g/L KH_2PO_4, 1 g/L NH_4Cl, 0.5 g/L NaCl, 1 mM $MgSO_4$, 0.1 mM $CaCl_2$
- add all supplements including nicotinic acid, $CaCl_2$ and $MgSO_4$ to the M9 salts after the autoclaving
- Concentrations:
  - threonine = 40 µg/ml
  - proline = 40 µg/ml
  - thiamine = 1 µg/ml
  - glucose = 0.2% w/v
  - kanamycin = 50 µg/ml

Pre-mating outgrowth:

*Donor- EcS17/pSAM-Ec***:**

Grow in 5 mL pre-mating media, shaking at 37°C in the presence of ampicillin (100 µg/ml) to an optical density at 600 nm (OD_{600}) of ~0.5 to 0.8. Collecting the donor strain after it reaches mid-log growth phase is optimal for mutagenesis. EcS17/pSAM-Ec needs ~36 to 48 h to reach an OD_{600} 1.0. To coordinate with the recipient pre-mating culture, subculture the donor strain (1:25, or 200 µl in to 5 ml) from a ~36 to 48 h culture into fresh pre-mating media plus ampicillin. It will then take ~7 h to reach the proper OD_{600} of 0.5 to 0.8.

Recipient- ExPEC strain F11:

In our lab we have mutagenized various *E.coli* pathogens and non-pathogens with the pSAM-Ec vector and EcS17, but efficiency varies, and optimization may be required for each strain. Grow the recipient strain in pre-mating media overnight, shaking at 37°C. In the morning, subculture the recipient strain into 5 ml fresh pre-mating media and culture until an OD_{600} of 0.5 to 0.8 is reached. Heat-shock the recipient for 30 minutes at 50°C.

Example protocol chronology:

Day 1:
1. In the morning, start a 5 mL culture of donor strain (EcS17/pSAM-Ec) in pre-mating media plus ampicillin.

Day 2:
1. Start overnight culture of recipient strain in pre-mating media.

Day 3:
1. Subculture donor strain into 5 mL pre-mating media plus ampicillin. Incubate about 7 hours to obtain OD_{600} of 0.5 to 0.8. Subculture the recipient strain so that it reaches a similar OD_{600} at the same time as the donor.

2. Heat shock recipient strain at 50° C for 30 minutes.

3. Measure OD_{600} of both the donor and recipient cultures. Dilute the recipient using M9 salts to an OD_{600} that is half the OD_{600} of the donor. This dilution is used to achieve a recipient cell concentration approximately half that of the donor.

4. Add 750 µl each of donor and recipient culture to a microcentrifuge tube to form the mating mixture (1.5 ml total volume).

5. Wash #1- pellet the mixture by centrifuging at high-speed, aspirate supernatant, resuspend in 500 µl M9 salts.

6. Wash #2- pellet the mixture by centrifuging at high-speed, aspirate supernatant, resuspend in 150 µl pre-mating media.

7. Place a sterile 0.45 µm membrane disc (Millipore, cat. no. HAWP02500) onto the surface of a mating agar plate. Apply 50 µl of the mating mixture to the surface of the membrane. Multiple matings can be performed from one mating mixture.

8. Incubate plates upright at 37°C for 5 hours.

9. Remove individual membranes from mating agar plates with sterile tweezers and place in a 50 mL conical tube containing 2 mL M9 salts. Dislodge bacteria by vortexing.

10. Gently centrifuge to collect all liquid in the bottom of the tube.

11. Serially dilute and plate 100 µl of the mating suspension on selective agar plates and incubate at 37°C overnight. If mutagenesis efficiency is high, may need to plate 10^{-1} and 10^{-2} dilutions. These plates will be used to estimate mutagenesis frequency and infer how many distinct mutants were generated.

12. Add the remainder of the mating suspension to a 250 mL flask containing 20 mL of selective media. Incubate shaking at 37°C for 40 to 60 min to allow transposon mutants time to express the kanamycin resistance gene. After this initial growth period, add the kanamycin antibiotic and continue to culture until an OD_{600} of 0.4 to 0.5 is reached. This normally takes about 2-3 h total.

13. Optional: Serially dilute and plate the mutant outgrowth culture onto Luria Bertani (LB) agar plates +/- kanamycin to estimate purity of the mutant library. We observed our mutant libraries to be nearly 100% pure using this procedure. Additionally, another optional serial dilution and plating can be done using LB agar plates +/- ampicillin to ensure that mutants no longer carry the pSAM-Ec plasmid.

14. Freeze down 1 mL freezer stocks—at least 1 stock for long-term storage/use and 1 axillary stock for thawing and combining with other mutant libraries. Mix 1 mL of culture and 0.5 mL 80% glycerol for storage at -80°C.

Day 4:

1. Count plates containing serial dilutions from the mating suspension (Day 3, step 11). Calculate mutagenesis frequency (transposon hops per mating). This number can be used to infer how many distinct transposon mutants are contained within each stock. NOTE: this calculation is purely an estimate and will likely differ after determination by deep sequencing. However, we did find this number was reliable for use in the design and execution of our selection screen.

This protocol was assembled and optimized with the help of Stephanie Aoki (David Low Lab, UC Santa Barbara) and Colin Russell (Matt Mulvey Lab, University of Utah).

Additional references:

Rubin EJ, *et al.*, 1999. *In-vivo* transposition of *mariner*-based elements in enteric bacteria and mycobacteria. *PNAS*, 96: 1645-1650.

Gonzalez P, *et al.*, 1999. Increasing DNA transfer efficiency by temporary inactivation of host restriction. *BioTechniques*, 26: 892-900.

Southern blotting

Isolation and digestion of Genomic DNA:

1. Individual transposon mutants were isolated and cultured overnight in 5 ml LB media.

2. The Wizard Genomic DNA Purification Kit (Promega) was used per manufacture's protocol to harvest DNA from 2 ml of bacterial culture. The optional steps were taken to improve DNA yield:

   A. Step 4- tubes were vortexed during protein precipitation for at least 20 seconds.
   B. Step 5- after addition of isopropanol, tubes were inverted several times until DNA was clearly visible and free of air bubbles.
   C. After the drying step, the DNA pellet was suspended in sterile H_2O for at least 30 min with occasional vortexing. Incubating at 65°C was also done to facilitate this process.

3. 7.5 µg of genomic DNA from individual transposon mutants were digested with the frequent cutter HindIII (New England Biolabs) for 2 to 3 h. Digestions were inactivated and frozen at -20°C for later use. Alternatively, transposon mutants were also digested with MmeI to excise the transposon. The procedures for MmeI digestion of genomic DNA were performed as in Goodman *et al.,* 2009- PMID: 19748469.

Generation of digoxigenin (DIG)-dUTP labeled probes:

A 501 bp internal portion of the kanamycin resistance gene from pSAM-Ec was amplified using a PCR DIG Probe Synthesis Kit (Roche, cat. no. 11 636 090 910). Primers used are described in Table S3.

Hybridization and detection:

1. 5 µg of digested genomic DNA was resolved on a ¼ inch thick, 0.8% agarose gel by gel electrophoresis.

2. Passive transfer of genomic DNA was performed with the following protocol at room temperature.

A. *Acid depurination*- soak gel with gentle rocking in 0.25 M HCl 2 x 5 min. NOTE: loading dye will shift from blue to greenish yellow.
B. *Rinse* gel briefly with deionized H_2O.
C. *Denaturation*- soak gel with gentle rocking in 0.4 N NaOH for 30 minutes.
D. *Rinse* gel briefly with deionized H_2O.
E. *Neutralize*- soak gel with gentle rocking in "Neutralization" solution. 2 x 15 minutes.
F. *Equilibrate*- soak gel with gentle rocking in 20x SSC 2 x 15 minutes.

RECIPES:

- **Depurination solution**: 21.6 ml concentrated HCl mixed to 1 L with H_2O (add acid to water)
- **Denaturing Solution**: 40 g solid NaOH with water to 250 ml to make 4 N. Dilute 1:10 for use.
- **Neutralizing solution**: 78.8 g Tris base, 87.6 g NaCl in 800 ml H_2O, pH to 7.4 using concentrated HCl. Qs to 1 L.
- **20x SSC**: 175.3 g NaCl, 88.2 g sodium citrate (trisodium salt). Qs to 1 L.

G. *Assemble transfer tower*-
   i. Pre-soak the 0.45 µm nylon Biodyne B membrane (PALL) in H_2O for 5 minutes.
   ii. Soak the nylon membrane in 20x SSC for 5 minutes.
   iii. Fill the glass reservoir to be used for passive transfer with 20x SSC so the bottom edges of the "wicking" or "3MM bridging" paper are submerged by about ¾ inch. Thoroughly wet the bridge paper and every additional blotter paper before addition to the tower with 20x SSC—make sure there are no air bubbles. Once assembled, wrap in saran wrap for stability and to maintain humidity. Transfer overnight at room temperature.
   iv. Transfer tower diagram.

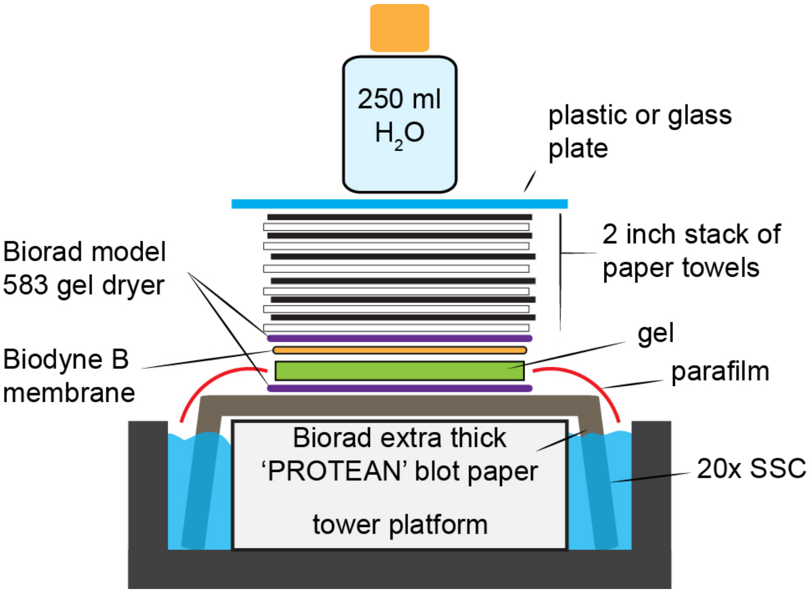

H. *Recovery of membrane-*

i. Recover nylon membrane and wash 2 x 15 minutes (DNA side up) in 2x SSC to remove debris and other fibers.

ii. Lay the membrane on a sheet of PROTEAN blot paper (Bio-Rad) to air dry. Change the blot paper after a few minutes and allow to completely dry at room temperature.

iii. Once dry, fix DNA to membrane by incubating for 30 minutes at 80°C between two sheets of PROTEAN blot paper to hold form.

I. *Pre-hybridization-*

i. Pre-warm 2x SSC and pre-hyb solution to 65°C.
Pre-hyb solution recipe: 10 µg/ml denatured salmon sperm (Sigma-Aldrich D9156) in PerfectHyb Plus (Sigma-Aldrich H7033)

ii. Pre-wet the membrane in 2x SSC.

iii. Remove 2x SSC and add pre-hyb solution to blot in a sealable K-pak bag. Rotate in rotisserie oven at 65°C for a minimum of an hour. Hybridize longer to reduce background. Use 100 µl hybridization solution/cm^2 blot.

J. *Hybridization-*

i. Denature the pre-made DIG probe for 5-10 minutes at 95°C and place immediately on ice.

ii. Add ~2 µl probe/ml pre-hyb solution plus salmon sperm (pre-warmed to 65°C).

iii. Pour off pre-hyb solution and add the probe/hyb mix. Hybridize overnight rotating at 65°C.

K. *Washes-*

i. Wash hybridized membrane 2 x 5 minutes at room temperature with 2x SSC (covered).
ii. Pre-warm the following solutions to 60°C for subsequent wash steps. (2x SSC plus 0.1% SDS) (1x SSC plus 0.1% SDS).
iii. Wash 2 x 15 minutes with 2x SSC/0.1% SDS at 60°C (covered).
iv. Wash 2 x 15 minutes with 0.1x SSC/0.1% SDS at 60°C (covered).

L. *Detection by enhanced chemiluminescence (ECL)-*
i. Block membrane for 2 to 4 h in 5% milk plus 0.1% SDS.
ii. Incubate membrane with anti-DIG antibody (Abcam) diluted 1:1000 in 5% milk plus 0.1% SDS overnight at 4°C.
iii. Wash membrane 4 x 5 minutes in TBS-t.
iv. Apply HRP-conjugated secondary antibody diluted 1:2,000 in 5% milk plus 0.1% SDS for 30 minutes at room temperature.
v. Wash blot 3 x 5 minutes in TBS-t followed by 2 x 5 minutes in TBS.
vi. Expose blot using the ECL Western Blotting System (Amersham).

Plating assays

Transposon mutant pools were grown for ~24 h in M9 media at 37°C. Each pool was then diluted and spread over MacConkey, Congo Red, and Kornberg agar plates. Bacteria were plated at a density that resulted in ~200-300 distinguishable colonies/plate. 5,000 to 9,000 individual colonies per pool per plating method were assessed.

Media recipes:

MacConkey Agar: MacConkey agar contains the sugar lactose as a principle carbon source. *E. coli* capable of fermenting this substrate lower the pH of their local environment. Because of the presence of the pH sensitive dye neutral red, colonies capable of lactose fermentation turn pink and colonies incapable remain colorless. MacConkey agar (Sigma-Aldrich) was prepared per manufacturer's instructions.

Congo Red Agar: The dye Congo red binds amyloid-like fibers, including the extracellular curli of *E. coli*. On agar media containing Congo red, curli-producing bacteria stain red whereas curli-negative bacteria remain colorless. Congo red agar plates were prepared by adding 0.001% Congo red dye to 1.2% LB agar media prior to pouring.

Kornberg Agar: Kornberg media containing 1% glucose was prepared as in Wei *et al*., 2000-PMID: 10692369. Growth on this medium induces glycogen storage within *E. coli*. After overnight growth or, until bacterial colonies are visible, the Kornberg agar plates are exposed to iodine vapor. Colonies with normal to excessive glycogen stain brown to black whereas colonies void of glycogen remain colorless.

<u>Preparation of excised transposons for indexed sequencing</u>

Genomic DNA was digested with the restriction endonuclease MmeI and was resolved by electrophoresis on a 1% agarose gel. A gel slice representing a size range from 1450 to 1650 bp was excised and purified using the Qiaquick Gel Extraction Kit (Qiagen). This size range enriched for fragments containing the transposable element and adjacent unique bases associated with the insertion site. End repair and dA tailing of the purified fragments was performed using the Illumina ChIP-Seq Sample Prep Kit. A double-stranded adapter sequence assembled by combining the following oligonucleotides, 5'-ACACTCTTTCCCTACACGACGCTCTTCCGATCT and 5'-GATCGGAAGAGCGTCGTGTAGGGAAAGAGTGT, was ligated to the ends of the adenylated DNA fragments. This adapter contains the appropriate DNA sequences to

enable priming of the first read on an Illumina sequencing flow cell, thus enabling unique sequences that are adjacent to either side of the transposable element to be identified by sequence analysis. Ligated fragments were purified using the Qiagen QIAquick PCR Purification Kit. Purified fragments were subjected to 8 cyles of PCR primed with the oligonucleotides 5'-AATGATACGGCGACCACCGAGATCTACACTCTTTCCCTACACGACGCTCTTCCGATCT and 5'-GTGACTGGAGTTCAGACGTGTGCTCTTCCGATCTCTTATCATCCAACCTGTTA. The PCR reaction separates the two ends of the transposable element from each other such that unique insertion site bases and 19 bp of transposable element sequence is positioned between Illumina adapter 1 and adapter 2. Eight additional cycles of PCR were performed to introduce a multiplexing index tag to each library member using an Illumina PCR primer index oligo along with the oligonucleotide 5'-AATGATACGGCGACCACCGAGA to prime amplification. PCR products were purified using a Qiagen QIAquick PCR purification kit and the libraries were validated by running an aliquot on an Agilent High Sensitivity DNA bioanalyzer chip and performing qPCR using the Kapa Biosystems Library Quantification Kit. Sequencing libraries were normalized to 10 nM and were pooled in equal volumes. Pooled sequencing libraries were sequenced on a 50 cycle single end read with an index read on an Illumina HiSeq 2000.
