# Supplementary material for: Combining Quantitative Genetic Footprinting and Trait Enrichment Analysis to Identify Fitness Determinants of a Bacterial Pathogen
Source: PLoS Genet. 2013 Aug 22;9(8):e1003716. doi: 10.1371/journal.pgen.1003716 (PMC3749937; doi:10.1371/journal.pgen.1003716)
Supplement: Table S1 — General sequence-based features of input and output pools. (PDF) [file pgen.1003716.s011.pdf]

Table S1. General sequence-based features of input and output pools

Replicate CFU/embryo	Pool	Detected mutant variants	Depth Sum of aligned reads	Saturation % of annotated genes with at least: 1 insert*	10 insertions*	Insertional bias % of inserts within annotated loci**
	Input	78,591	8,206,447	99.90	98.96	87.83
1 (2,000)	Blood	23,756	7,075,904	95.07	48.85	88.08
	PC	38,936	7,671,459	98.08	72.14	88.01
	Input	81,096	8,494,073	99.77	98.92	87.84
2 (3,000)	Blood	31,871	7,049,106	96.20	60.67	88.22
	PC	38,842	9,052,580	97.55	70.20	88.42
	Input	63,184	8,113,311	99.54	99.00	88.17
3 (4,000)	Blood	31,927	5,899,109	96.69	62.41	87.78
	PC	44,926	7,309,071	97.93	73.21	88.07

*values reflect proportion of 5,312 annotated regions within the F11 genome.

**4,628,529 bp (out of 5,215,961 bp) are contained within annotated F11 loci; therefore, it is expected that the percent of inserts found within annotated regions would be 88.73%.
