# Supplementary material for: Combining Quantitative Genetic Footprinting and Trait Enrichment Analysis to Identify Fitness Determinants of a Bacterial Pathogen
Source: PLoS Genet. 2013 Aug 22;9(8):e1003716. doi: 10.1371/journal.pgen.1003716 (PMC3749937; doi:10.1371/journal.pgen.1003716)
Supplement: Table S4 — Composition of TEA-defined pathogen-associated genes within Tn-seq gene sets. (PDF) [file pgen.1003716.s014.pdf]

Table S4. Composition of TEA-defined pathogen-associated genes within Tn-seq gene sets.

	**Gene Set** (total genes)	**observed** (% of gene set)	**theoretical**	***p* value**
	F11 genome (5,148)	1,166 (22.65)	-	-
	Hypo-tolerant (609)	120 (19.70)	127.84	0.803
	Hyper-tolerant (431)	81 (18.79)	91.06	0.899
■	*in piscis* (970)	246 (25.36)	218.11	0.009
	Multi-niche (76)	15 (19.74)	17.31	0.786
■	PC (122)	39 (31.97)	27.52	0.008
■	Blood (772)	192 (24.87)	173.54	0.0485
■	Advantageous (227)	69 (30.39)	50.46	0.004

Significant enrichment of pathogen-associated genes within Tn-seq gene sets was detected using 1,000 Monte Carlo simulations. For the ‘Blood’ gene set, 10,000 simulations were performed to generate a more discernable *p* value. Significant enrichments ($p < 0.05$) are marked with a blue box.
