# Supplementary material for: Combining Quantitative Genetic Footprinting and Trait Enrichment Analysis to Identify Fitness Determinants of a Bacterial Pathogen
Source: PLoS Genet. 2013 Aug 22;9(8):e1003716. doi: 10.1371/journal.pgen.1003716 (PMC3749937; doi:10.1371/journal.pgen.1003716)
Supplement: Table S5 — Bacterial strains and plasmids. (PDF) [file pgen.1003716.s015.pdf]

Table S5. Bacterial strains and plasmids.

Strain or Plasmid	Description	Reference/Source
E. coli		
F11	ExPEC (human cystitis isolate, O6:K2:H31)	[1,2]
EcS17	pir^{+} donor strain	[3,4]
Plasmids		
pKM208	Encodes IPTG-inducible lambda Red recombinase; Amp^r	[5]
pKD4	Template source for kanamycin resistance cassette; Kan^r	[6]
pGFP-Mut3.1	Source vector for P_{lac} promoter; Amp^r	Clonetech
pSAM_Bt	Source plasmid for construction of pSAM-Ec; Amp^r, Erm^r	[4]
pSAM-Ec	Contains the *himar*1C9 transposase gene driven by a P_{lac} promoter and a *mariner* transposon encoding a kanamycin resistance gene; Amp^r, Kan^r	This study
Recombinant strains		
F11/pKM208	F11 with pKM208; Amp^r	This study
F11Δ*3256*	F11 *EcF11_3256* (*typA*/*bipA*)::*kan*; Kan^r	This study
F11Δ*3082*	F11 *EcF11_3082* (*mprA*/*emrR*)::*kan*; Kan^r	This study
F11Δ*2628*	F11 *EcF11_2628* (hypothetical)::*kan*; Kan^r	This study
F11Δ*3933*	F11 *EcF11_3933* (hypothetical)::*kan*; Kan^r	This study

References

1. Lloyd AL, Rasko DA, Mobley HL (2007) Defining genomic islands and uropathogen-specific genes in uropathogenic Escherichia coli. Journal of bacteriology 189: 3532-3546.
2. Wiles TJ, Bower JM, Redd MJ, Mulvey MA (2009) Use of zebrafish to probe the divergent virulence potentials and toxin requirements of extraintestinal pathogenic Escherichia coli. PLoS pathogens 5: e1000697.
3. Cowles CE, Nichols NN, Harwood CS (2000) BenR, a XylS homologue, regulates three different pathways of aromatic acid degradation in Pseudomonas putida. Journal of bacteriology 182: 6339-6346.
4. Goodman AL, McNulty NP, Zhao Y, Leip D, Mitra RD, et al. (2009) Identifying genetic determinants needed to establish a human gut symbiont in its habitat. Cell host & microbe 6: 279-289.
5. Murphy KC, Campellone KG (2003) Lambda Red-mediated recombinogenic engineering of enterohemorrhagic and enteropathogenic E. coli. BMC molecular biology 4: 11.
6. Datsenko KA, Wanner BL (2000) One-step inactivation of chromosomal genes in Escherichia coli K-12 using PCR products. Proceedings of the National Academy of Sciences of the United States of America 97: 6640-6645.
