# Supplementary material for: Combining Quantitative Genetic Footprinting and Trait Enrichment Analysis to Identify Fitness Determinants of a Bacterial Pathogen
Source: PLoS Genet. 2013 Aug 22;9(8):e1003716. doi: 10.1371/journal.pgen.1003716 (PMC3749937; doi:10.1371/journal.pgen.1003716)
Supplement: Table S6 — Primers used in this study. (PDF) [file pgen.1003716.s016.pdf]

Table S6. Primers used in this study.

Primer Use/Name	Sequence 5'-3'
**Cloning**	
P_{lac} promoter	
P_{lac}_BamHI_Forward	GTAGGATCCAGTGAGCGCAACGCAATTA
P_{lac}_NdeI_Reverse	GTACATATGTATCCGCTCACAATTCCACA
Kanamycin^r cassette	
Kan_MfeI_Forward	TCGCAATTGAGGAAGCGGAACACGTAGAA
Kan_XbaI_Reverse	TCGTCTAGAGTCATTTCGAACCCCAGAGTC
pSAM promoter sequencing	
-69_Forward	TGGCCTTTTTGCGTTTCTAC
+65_Reverse	CAAGCCAAGTTTTTGCTTCC
**Southern blot probe**	
Kanamycin gene	
SB_probe_Forward	AGGAAGCGGAACACGTAGAA
SB_probe_Reverse	CTCGTCCTGCAGTTCATTCA
**Knockout and confirmation**	
EcF11_3256 KO	
Forward	TGTACAATAACGCGCTATTTCTAATGCCTGAGGCAAAGTTTGTGTAGGCTGGAGCTGCTTCG
Reverse	CTGCCATCGCTGGCAGGTTTTTTATGACTAAAAAACGAAACATATGAATATCCTCCTTAG
EcF11_3256 KO confirm	
Forward	CAATACTTGTTTGCGGAGGA
Reverse	GGGAGATACTCGCATTGGTG
EcF11_3082 KO	
Forward	TCGTTACTATATCGGCTGAAATTAATGAGGTCATACCCAATGTGTAGGCTGGAGCTGCTTCG
Reverse	TTTTCTTTTATAAATCTGGATTTTTGAGCGAGATGACGCGCATATGAATATCCTCCTTAG
EcF11_3082 KO confirm	
Forward	TCGGCTGAAATTAATGAGGTC
Reverse	CCAAAAAGGCCAGCATGT
EcF11_2628 KO	
Forward	TAGCTCTACCACGTATTTTTTTTCTAAACATTATCTACCTTGTGTAGGCTGGAGCTGCTTCG
Reverse	TATTCCTAATATGGCAACTTTAAGTTATCGCCGTCTATACCATATGAATATCCTCCTTAG
EcF11_2628 KO confirm	
Forward	CAGGATTTTTGCTAACATAACCTT
Reverse	GGCGCACCGTGTTCTTTC
EcF11_3933 KO	
Forward	TTTATCCTCTTGCCCTTGCTTCTACCTCGGTAAAAAATTGTGTGTAGGCTGGAGCTGCTTCG
Reverse	AAACAGGTGTTGCACACAACCAGACGCGGCACCTGTTTACATATGAATATCCTCCTTAG
EcF11_3933 KO confirm	
Forward	GCATGGACGCTGACAGTTC
Reverse	TGTGGATGAAGGGGTAAGGT
