# Supplementary material for: Combining Quantitative Genetic Footprinting and Trait Enrichment Analysis to Identify Fitness Determinants of a Bacterial Pathogen
Source: PLoS Genet. 2013 Aug 22;9(8):e1003716. doi: 10.1371/journal.pgen.1003716 (PMC3749937; doi:10.1371/journal.pgen.1003716)
Supplement: Table S8 — TEA-assigned trait identities for known bacterial virulence factors and conserved genes involved in basic bacterial physiology. (PDF) [file pgen.1003716.s018.pdf]

Table S8. TEA-assigned trait identities for known bacterial virulence factors and conserved genes involved in basic bacterial physiology.

	protein	function	F11 Locus_Tag	number of TEA-MD bacteria with homologues (% pathogens)	significance of pathogen association (*p* value)
virulence genes	FyuA	iron acquisition	EcF11_3031	36 (69.4)	0.010
	FepA	iron acquisition	EcF11_0158	51 (62.7)	0.029
	HlyA	toxin	EcF11_0729	8 (100)	0.002
	VasA	type VI secretion	EcF11_3189	39 (71.7)	0.003
	ClpV	type VI secretion	EcF11_3195	40 (65)	0.024
	PapG	adhesion	EcF11_0768	8 (100)	0.004
	Tsh	adhesion	EcF11_3654	15 (93.3)	<0.001
	KpsC	capsule	EcF11_1397	17 (82.3)	0.007
conserved genes	FimH	adhesion	EcF11_0633	29 (62)	0.145
	Crp	carbon utilization	EcF11_4019	102 (46)	0.952
	BipA	translation	EcF11_3256	159 (50.9)	0.620
	LrhA	transcriptional	EcF11_1255	70 (52.8)	0.387
	CheY	chemotaxis	EcF11_4121	69 (53.6)	0.334
	OmpR	osmoregulation	EcF11_3975	85 (51.7)	0.477
	OxyR	oxidative stress response	EcF11_2992	112 (51.7)	0.422
	RpoE	envelope stress response	EcF11_4796	113 (48.6)	0.840
