# Supplementary material for: Correction: Combining Quantitative Genetic Footprinting and Trait Enrichment Analysis to Identify Fitness Determinants of a Bacterial Pathogen
Source: PLoS Genet. 2013 Sep 6;9(9):10.1371/annotation/6bb09d48-7d06-4ccf-8ed1-3dfaa1e0d537. doi: 10.1371/annotation/6bb09d48-7d06-4ccf-8ed1-3dfaa1e0d537 (PMC3795158; doi:10.1371/annotation/6bb09d48-7d06-4ccf-8ed1-3dfaa1e0d537)
Supplement: Supplementary file 1 [file pgen.6bb09d48-7d06-4ccf-8ed1-3dfaa1e0d537.s001.pdf]

Description of pipeline used to define Tn-seq candidate gene sets.

I normalize insert read counts

For each input/output pool pair (e.g., input pool 1 and blood pool 1), a relative occurrence frequency was derived by normalizing insert counts to the sum of all aligned reads that were detected in both samples.

II identify strong negative selection events and median center output data

Because our insert tracking approach initially required inserts to be detected in both input and cognate output samples, we needed to account for inserts that had been completely lost due to strong negative selection. We reasoned that if *lost* inserts had a high enough input occurrence frequency, their absence from output pools likely reflected a true negative selection event. Therefore, we included *lost* inserts in our analysis by assigning them a limit of detection value if they had an input occurrence frequency within the top 1% of all frequencies of *lost* inserts. This input occurrence frequency cutoff corresponded to 2 to 3 times the median input occurrence frequency for *found* inserts. All output occurrence frequencies were then median-centered to account for global, insert-independent phenomena generated by experimentally introduced bottlenecks.

III perform Wilcoxon signed-rank test

Using the paired insert data from step **II**, a Wilcoxon signed-rank test was used to determined if the distribution of insert occurrence frequencies within a given region significantly differed between input and output samples. The Wilcoxon signed-rank test was chosen because of its conservative nature and its relative resistance to spurious signals from outliers.

IV initial candidate list vetting

Three parameters were used to generate the gene sets '*in piscis*' and 'advantageous'. If a gene had a Wilcoxon p value < 0.05, 10 or more measured inserts, and averaged at least a 2-fold decrease in insert abundance compared to output samples, it was assigned to the '*in piscis*' gene set. If a gene met the same significance and insert cutoffs, but exhibited at least a 2-fold increase in insert abundance compared to output samples, it was assigned to the 'advantageous' gene set.

V final gene set construction and curation

Using insertion data from input pools, genes were assigned to 'hypo-tolerant' or 'hyper-tolerant' gene sets if they tolerated a relatively low ($Z < -1$) or high ($Z > 1$) number of insertion events, respectively. The use of standard scores provided an intuitive demarcation of the 'hypo-tolerant' and 'hyper-tolerant' gene sets that was corroborated by functional analysis using KEGG (Figure 3). Considering a single infection site, if a gene was assigned to both the '*in piscis*' and 'advantageous' gene sets, it was subtracted from both. The '*in piscis*' gene set was further divided into the component gene sets 'multi-niche' (genes that confer fitness in the PC and blood), 'PC' (genes that confer fitness in the PC only) and 'blood' (genes that confer fitness in the blood only). False discovery rate for both '*in piscis*' and 'advantageous' gene sets was calculated to be <10%.
